# Metabolic inhibition of glutamate-cysteine ligase increases dendritic cell-mediated antitumor immunity in melanoma

**DOI:** 10.1136/jitc-2026-014808

**Published:** 2026-06-30

**Authors:** Sophie Marie Dieckmann, Christoph H Tripp, Helen Strandt, Florian Hornsteiner, Annelie Kerstin Schäfer, Anastasia Prokopi, Janine Vierthaler, Antonia Resag, Daniela Ortner-Tobider, Vera Reinstadler, Irene Rigato, Berit Junger, Alexeja Kleiter, Georgios Fotakis, Mirjana Efremova, Zlatko Trajanoski, Louis Boon, Suzie Chen, Anja Katrin Bosserhoff, Herbert Oberacher, Francesca Finotello, Patrizia Stoitzner

**Affiliations:** 1 Department of Dermatology, Venereology and Allergology, Medical University of Innsbruck, Innsbruck, Austria; 2 Department of Internal Medicine V, Medical University of Innsbruck, Innsbruck, Austria; 3 Tyrolean Cancer Research Institute, Medical University of Innsbruck, Innsbruck, Austria; 4 Centre for Immunobiology and Infection, Blizard Institute, Barts and the London School of Medicine and Dentistry, Queen Mary University of London, London, UK; 5 Department of Dermatology, Venerelogoy and Allergology, Medical University of Innsbruck University Hospital for Dermatology and Venereology, Innsbruck, Austria; 6 Institute of Immunology, Dresden University of Technology, Dresden, Germany; 7 Institute of Legal Medicine and Core Facility Metabolomics, Medical University of Innsbruck, Innsbruck, Austria; 8 Department of Molecular Biology, Digital Science Center (DiSC), University of Innsbruck, Innsbruck, Austria; 9 Department of Pediatrics I, Medical University of Innsbruck, Innsbruck, Austria; 10 Institute of Bioinformatics, Medical University of Innsbruck, Innsbruck, Austria; 11 Barts Cancer Institute, Queen Mary University of London, London, UK; 12 Biocenter, Institute of Bioinformatics, Medical University of Innsbruck, Innsbruck, Austria; 13 JJP Biologics, Warsaw, Poland; 14 Department of Chemical Biology, Rutgers The State University of New Jersey, New Brunswick, New Jersey, USA; 15 Institute of Biochemistry, FAU, Erlangen, Germany

**Keywords:** Melanoma, Dendritic Cells, Tumor microenvironment - TME, Immunotherapy

## Abstract

**Background:**

Metabolic competition and nutrient restriction in the tumor microenvironment (TME) shape the immune infiltrate in tumors and subsequently tumor immunity. In this study we used the transgenic melanoma mouse model tg(Grm1)EPv, which spontaneously develops melanoma due to the ectopic expression of the metabotropic glutamate receptor 1 (*Grm1*) in melanocytes to investigate if aberrant glutamate metabolism drives tumor formation and affects immune cell function.

**Methods:**

We performed liquid chromatography-tandem mass spectrometry (LC-MS/MS)-based metabolomic analyses and RNA sequencing on tumor-free and tumor-bearing tg(Grm1)EPv tissues to characterize metabolic alterations associated with tumor progression. Flow cytometry was used to examine changes in immune cell subsets within the TME. To assess the functional relevance of glutamate metabolism, we inhibited glutathione metabolism using L-buthionine-(S,R)-sulfoximine (BSO), an inhibitor of glutamate-cysteine ligase that depletes cellular glutathione levels.

**Results:**

LC-MS/MS-based metabolomic analyses and RNA sequencing revealed changes in glutamate and glutamine metabolism, a glycolytic shift (Warburg effect), and reduced ATP levels in advanced tumors compared with tumor-free tissue, suggesting respiratory chain dysfunction. These metabolic changes in the TME are advantageous for the tumor cells and unfavorable for immune cells, such as dendritic cells (DC). Indeed, flow cytometry analysis of myeloid subsets during tumor progression showed a decline in tumor-infiltrating conventional type 2 DC and macrophages, alongside an increase in neutrophil and monocyte populations in advanced lesions. Interference with glutamate metabolism using BSO induced immunogenic cell death, namely ferroptosis, in an tg(Grm1)EPv-derived cell line in vitro. Therefore, we evaluated the combination of this inhibitor with immunotherapy as a promising new approach for the treatment of tumors in the tg(Grm1)EPv mouse model. We observed that tumor growth could be delayed in vivo when BSO was combined with a therapy regimen boosting DC numbers and activation. This inhibition of tumor growth was supported by the infiltration of activated T cells.

**Conclusion:**

Overall, our findings provide novel insights into the importance of combining metabolic intervention with immunotherapy for the treatment of patients with melanoma, particularly those bearing glutamate pathway-active or immunologically cold tumors. This knowledge can drive the design of novel therapeutic strategies for patients with cancer.

**WHAT IS ALREADY KNOWN ON THIS TOPIC**

- Several studies demonstrated that melanoma cells rewire their metabolism to favor oxidative phosphorylation, glycolysis, and glutaminolysis. Metabotropic glutamate receptor 1 (*Grm1*)-expressing melanoma cells increase their glutamate release, which might contribute to an immunosuppressive tumor microenvironment that impairs dendritic cell (DC) function.

**WHAT THIS STUDY ADDS**

- Our study demonstrates that melanoma progression in the *Grm1*-expressing EPv mouse model is accompanied by enhanced glycolytic activity and increased glutaminolysis. We identified L-buthionine-(S,R)-sulfoximine (BSO) in combination with DC-boosting immunotherapy as a promising combination approach that significantly delays tumor growth in vivo. This highlights a functional link between tumor metabolic pathways and immune activation.

**HOW THIS STUDY MIGHT AFFECT RESEARCH, PRACTICE OR POLICY**

- The findings reveal that targeting glutamate metabolism can sensitize melanoma to immune-based therapies, offering a rational strategy for improving outcomes in tumors resistant to immune checkpoint blockade. This work encourages future research into combined metabolic and immunotherapeutic approaches and supports the exploration of metabolic inhibitors as adjuncts to clinical immunotherapy protocols for melanoma and potentially other cancers.

## Introduction

 Melanoma represents a very aggressive type of skin cancer with a high mortality rate. Although the introduction of immune checkpoint blockade (ICB) therapy has significantly improved clinical outcomes with response rates of up to 50%, a large portion of patients do not respond to this first-line treatment, and novel therapeutic strategies are urgently needed.^1^,^3^

Metabolic reprogramming is recognized as one of the hallmarks of cancer, essential to meet the altered energy requirements for uncontrolled tumor cell proliferation.^4^ Melanoma cells suppress the tricarboxylic acid (TCA) cycle and melanogenesis while enhancing mitochondrial oxidative phosphorylation (OxPhos), glycolysis, the pentose phosphate pathway, and fatty acid synthesis to support rapid cell division.^4^ Furthermore, melanoma cells increase their glutamine uptake and glutaminolysis to produce glutamate via glutaminase (GLS).^5^ An overexpression of glutamate receptors and elevated glutamate levels have been described in several cancer types, for example, the metabotropic glutamate receptor 1 (Grm1) drives tumor growth by an autocrine loop in melanoma, breast cancer, prostate cancer, and lung cancer.^6^,^8^

In the autochthonous tg(Grm1)EPv melanoma mouse model (hereafter referred to as EPv), the ectopic expression of Grm1 under a melanocyte-specific promoter causes spontaneous melanoma development in the skin of the ears, tail, and perianal area, as melanocytes are distributed in an interfollicular pattern in these skin areas.^9^ Grm1 is a seven-transmembrane-domain receptor that belongs to the G-protein-coupled receptor superfamily. On binding of glutamate, the activation of the mitogen-activated protein kinase (MAPK) signaling cascade drives cell proliferation and inhibits apoptosis, and PI3K/AKT pathway activation supports tumor cell survival, epithelial–mesenchymal transition, and angiogenesis.^10 11^ Aberrant *GRM1* expression can be found in more than half of patient melanoma samples, while human melanocytes are *GRM1*-negative, suggesting an involvement of *GRM1* in the development of human melanoma.^12^

Metabolic competition and nutrient restriction in the tumor microenvironment (TME) not only impact the tumor cells themselves but can also modulate the immune infiltrate and subsequently tumor immunity.^13^ Dendritic cells (DCs) play an important role in the induction of antitumor immune responses as they are equipped with the unique ability to cross-present tumor-derived antigens, driving T cell but also natural killer (NK) cell responses for successful immunotherapy.^14^ The DC family consists of several subsets, such as conventional DC type 1 (cDC1) and cDC2, plasmacytoid DC (pDC), and Langerhans cells (LCs) in the skin.^15^ Lactate, a by-product of glycolysis that can be derived from melanoma cells, remodels the metabolic profiles of DC subsets.^16^ It can block antigen presentation by DC in the TME as well as their expression of costimulatory molecules and DC migration to lymph nodes (LN).^17^ High levels of reactive oxygen species (ROS) in the TME promote lipid peroxidation and trigger IRE-XBP1 activation in DC, which subsequently inhibits T cell activation.^18^ The release of glutamate during glutaminolysis in the immunological synapse is important for T cell activation,^19^ and the amino acid transporters SLC1A5 and SLC38A2 regulate immune infiltrates in the TME.^20^

We previously reported that during tumor progression in the EPv mouse model, late-stage tumors were dominated by immunosuppression, as indicated by the infiltration of myeloid-derived suppressor cells (MDSC), loss of DC, and impaired T cell function.^21 22^ Tumor growth could be delayed with the combination of ICB antibodies and a regimen that enhanced DC numbers and function, confirming the importance of DC for successful immunotherapy in melanoma.^22^ As combinations of metabolic inhibitors with immunotherapy are promising new approaches for the treatment of patients with cancer refractory to standard therapies, in this study we aimed to investigate the metabolic changes during tumor development in the transgenic EPv melanoma mouse model. We observed that melanoma development was accompanied by increased aerobic glycolysis and glutaminolysis. Interference with glutamate metabolism using L-buthionine-(S,R)-sulfoximine (BSO), an inhibitor of the glutamate-cysteine ligase (GCLC), induced programmed cell death, namely ferroptosis, in an EPv-derived cell line in vitro. Tumor growth could be delayed in vivo when BSO was combined with DC boost therapy.

## Materials and methods

### Human melanoma transcriptomic survival analysis

Clinical metadata and transcriptomic expression data for skin cutaneous melanoma (SKCM) samples (n=470) were obtained from The Cancer Genome Atlas (TCGA) using the associated GDC data portal (https://portal.gdc.cancer.gov/). Clinical records were cleaned, and overall survival time and event status were computed from available follow-up and mortality fields.

A predefined glutaminolysis-related gene signature (*GLS, GSS, SLC3A2, SLC7A11, GLUD1, SLC1A5, SLC6A15, SLC38A5, GLUD2, SLC38A2*^23^) was scored using gene set variation analysis (GSVA). The resulting enrichment scores were merged with matching clinical metadata. Optimal cut-points separating high versus low signature expression were determined for both the GSVA results and the *GRM1* gene expression using maximally selected rank statistics (surv_cutpoint() function from the package survival V.3.8-3). Kaplan-Meier survival models were then generated using survival (survival V.3.8-3) and survminer (survminer V.0.5-1) in R V.4.4.2, stratified by high versus low signature activity. Survival curves were plotted with p values and median survival estimates.

### Mice

The tg(Grm1)EPv (EPv) mice (kindly provided by Jürgen C Becker, University of Duisburg-Essen) were established on a C57BL/6J background at the Department of Chemical Biology, Rutgers University. They develop spontaneous tumors predominantly in the ear and tail skin due to melanocyte-specific dopachrome tautomerase promoter-driven ectopic expression of the Grm1 transgene.^9 21^ The classification of the mice in different tumor stages was performed in the following way: mice were classified as tumor-free (TF) at the age of 0–10 weeks (100–200 µm ear thickness, 0–50 mg ear weight), and were compared with tumor-early (TE) at 3–6 months (200–700 µm ear thickness, 50–120 mg ear weight) and to tumor-advanced (TA) at 7–10 months (700–2000 µm ear thickness, 120–300 mg ear weight) mice.

### Tumor growth measurements

Ear thickness measurements for tumor growth monitoring were conducted using a caliper (Kroeplin, Schluchtern, Germany). A total of six measurements per mouse per time point were taken (three per each ear) and based on these the average ear thickness was calculated. Tumors in the EPv mouse model develop spontaneously and therefore can vary significantly in size regardless of age. For this reason, tumor growth alterations (increase/decrease) were calculated from raw values of ear thickness and in some cases normalized to the start of the experiment and shown as plus/minus the raw values of Δear thickness measured every week.

### Preparation of tissue samples for metabolite quantification

One ear per mouse was harvested and immediately transferred into a precooled 14 mL polystyrene Falcon tube. Each sample was weighed using an analytical balance, after which 1 mL of extraction solvent consisting of 80% methanol (v/v) was added. Each sample was precut into small fragments using sterilized scissors to facilitate homogenization. Further homogenization was performed using a Tissue Ruptor (Omni TH Tissue Homogenizer) at medium speed until uniform consistency was achieved, typically within 30–60 s. Homogenized samples were transferred into precooled 1.5 mL Eppendorf tubes and the homogenates were centrifuged at 10,000 × g for 10 min at 4°C to remove debris. The clear supernatant was collected and stored at −80°C until analysis.

### Mass spectrometric analysis of intermediates of glycolysis and TCA cycle

Find the chemicals used in online supplemental table T1.

Aliquots of cell lysates (50 µL) were mixed with 2.5 µL internal standard solution (5 µg/mL each). The liquid was evaporated at 50°C under a gentle stream of nitrogen. The dried residue was dissolved in 50 µL of 80% acetonitrile (v/v) containing 1 µM EDTA and submitted to liquid chromatography-tandem mass spectrometry (LC-MS/MS) analysis. The chromatographic system consists of an Agilent 1100 Series HPLC (Agilent, Santa Clara, California, USA) and an HTC PAL autosampler equipped with a Stack Cooler DW (both CTC Analytics AG, Zwingen, Switzerland). The autosampler tray temperature was set to 10°C. The injection volume was 10 µL. Mobile Phase A was an aqueous 10 mM ammonium acetate solution with 2.5 µM medronic acid at pH 9. Mobile Phase B consisted of 10 mM ammonium acetate in water/acetonitrile 15:85 (v/v) with 2.5 µM medronic acid at pH 9. Chromatographic separation was performed on an Agilent InfinityLab Poroshell 120 HILIC-Z column (2.7 µm, 2.1×100 mm, PEEK lined; Agilent), protected by an InfinityLab Poroshell 120 HILIC-Z guard column (2.7 µm, 3.0×5 mm), by applying a gradient of 96–6% B within 7 min, an isocratic period for 1 min with 6% B, followed by a gradient of 6–96% B within 1 min and 6 min re-equilibration time at 96% B. The flow rate was set to 250 µL/min and the total run time was 15 min. The column temperature was held at 30°C. The column outlet was directly connected to the mass spectrometer. Find the chemicals used in online supplemental table T1. Mass spectrometric detection was performed on a 4000 QTrap mass spectrometer (Sciex, Framingham, Massachusetts, USA) equipped with a Turbo V Ion Source operated in negative ESI mode. The spray voltage was set to −4500 V, gas flows of 60 arbitrary units were employed for both the nebulizer gas and turbo gas. The temperature for the turbo gas was set at 450°C and the collision gas was adjusted to medium. The mass spectrometer was controlled with the software Analyst 1.5.1 (Sciex). Compound-specific ion transitions and corresponding experimental parameters are summarized in online supplemental table T2. A 10-point calibration series was used for quantification (0.1−100 ng/mL). The peak ratios of analytes to internal standards were used to fit 1/x-weighted linear, least squares regression models. Measured metabolite concentrations were normalized to TF (healthy tissue) (glycolysis, TCA cycle) to further understand the metabolic changes occurring during tumor progression.

### Mass spectrometric analysis of amino acids

Find the chemicals used in online supplemental table T1.

2.5 µL of the cell lysates or 10 µL of calibration standards were pipetted into a 1.5 mL vial and mixed with 5 µL of the internal standard solution (2.5 µM of each internal standard in methanol-water (1:1, v/v)) as well as 65 µL methanol, 5 µL water, 10 µL triethylamine and 10 µL phenyl isothiocyanate. Derivatization was performed at room temperature for 30 min, after which, the excess reagent was removed by drying at 60°C with a gentle stream of nitrogen. The dried samples were dissolved with 100 µL of 25% methanol (v/v). The LC-MS/MS system consisted of Acquity UPLC H-Class Plus Bio System (Waters, Milford, Massachusetts, USA) and a QTrap 6500+mass spectrometer (Sciex). Separations were accomplished on an Eclipse XBD-C18 column (3.5 µm, 3.0×100 mm, Agilent) using a 10 min gradient of 2–98% acetonitrile in aqueous 0.5% acetic acid solution. The flow rate was set to 250 µL/min, and the column temperature was kept at 50°C. The injection volume was 10 µL. Mass spectrometry detection was performed with electrospray ionization in positive ion mode. Multiple reaction monitoring was carried out using the precursor-to-product ion transitions summarized in online supplemental table T3. The peak area ratios of analyte-specific ions to the corresponding internal standard ions obtained from the calibration standards versus concentrations were used to fit 1/x−weighted linear, least squares regression models. The calibration models were used to calculate the analyte concentrations from the peak area ratios of analyte-specific ions to the corresponding internal standard ions obtained from the samples. Measured metabolite concentrations were normalized to TF (healthy tissue) to further understand the metabolic changes occurring during tumor progression.

### Preparation of single cell suspensions from tumors and tumor-draining lymph nodes

Tumor-draining LN were dissected from mice, mechanically disrupted and digested with 250 µg/mL Collagenase D (Roche, Basel, Switzerland) and 120 µg/mL DNase I (Roche) in Hank’s Salt Solution (without Mg^2+^ and Ca^2+^, PAN-Biotech, Aidenback, Germany) supplemented with 2% fetal-calf serum (FCS). Tumor-draining LN were digested for 25 min at 37°C. Ear skin and ear tumors of EPv mice were separated into ventral and dorsal parts and then cut into small pieces in Hanks’ Salt Solution (with Ca^2+^ and Mg^2+^, Biochrom, Berlin, Germany). Then the tissue was enzymatically digested with 150 µg/mL Liberase (Roche Diagnostics, Mannheim, Germany) and with 120 µg/mL DNase (Sigma-Aldrich, Steinheim, Germany) at 37°C for 45 min. Digestion was stopped with 10 mM EDTA (Lonza, Basel, Switzerland) and tissue pieces were pressed through a 100 µm cell strainer (Corning, New York, USA) with a 2 mL syringe plunger (BD Biosciences, Franklin Lakes, New Jersey, USA) to obtain single cell suspensions for flow cytometry analysis.

### Flow cytometry analysis of mouse skin, tumors and LN

Cells were stained for 3 min at room temperature with the fixable viability dye eFluor780 (Thermo Fisher Scientific) to exclude dead cells, followed by incubation for 15 min with anti-mouse CD16/CD32 monoclonal antibody (mAb) (clone: 2.4G2, TONBO Biosciences, San Diego, California, USA) to prevent non-specific FcR-mediated antibody staining. Single cell suspensions were incubated with a mix of fluorescently labeled monoclonal antibodies for 30 min at 4°C. Surface staining for chemokine receptors CCR2 and CCR7 was performed for 30 min at 37°C. Following the surface staining, cells were washed twice and analyzed directly or used for intracellular staining. For this purpose, cells were fixed and permeabilized using the Cytofix/Cytoperm Kit (BD Biosciences) according to the manufacturer’s protocol. To analyze tumor necrosis factor-alpha (TNF-α) and interferon-gamma (IFN-γ) production, up to 1.5×10^6^ cells were restimulated in RPMI medium (Lonza, Switzerland) supplemented with 10% heat-inactivated FCS (PAN-Biotech), 50 units/mL Penicillin/Streptomycin (Life Technologies, New York, USA) and 2 mM L-glutamine (Lonza, Maryland, USA) with PMA (50 ng/mL, Sigma-Aldrich) and ionomycin (1 µg/mL, Sigma-Aldrich) for 4 hours in the presence of Brefeldin A (Thermo Fisher Scientific). For intracellular FoxP3 staining, cells were fixed and permeabilized for 45 min using the FoxP3 staining buffer kit according to the manufacturers’ protocol (eBioscience, Carlsbad, California, USA).

Flow cytometry sample acquisition was performed on a 5L Aurora Spectral Flow Cytometer (Cytek Bioscience, Amsterdam, Netherlands) and a CytoFLEX S (Beckman Coulter Life Sciences, Krefeld, Germany). Flow cytometry data was analyzed using FlowJo V.9 and V.10 (BD Biosciences) and in the cloud-based OMIQ analysis platform (Omiq, Boston, Massachusetts, USA). See full antibody list in the online supplemental table T4.

### High-dimensional flow cytometry analysis

For dimensionality reduction of tumor-infiltrating myeloid cells using uniform manifold approximation and projection (UMAP) and FlowSOM clustering analysis,^24^ data analysis pipelines using OMIQ were built. Data from tumor tissue was cleaned by removing cellular debris, doublets and dead cells and gated on viable CD45^+^ cells using FlowJo. Next, NK cells, T cells and B cells were analyzed separately, based on the gating strategy shown in the online supplemental figure S3. Files containing the myeloid cells of interest were exported from FlowJo and used for further analysis using OMIQ. Data was arcsinh transformed (cofactor 6000). All fluorescence parameters, excluding viability dye eFluor780, CD45 BUV805, lymphoid cell markers (NK1.1 BB630, CD3 PE-Cy5, CD19 BB660) and markers for phenotypical characterization of myeloid cells (CD40 BB700, CCR7 APC, programmed cell death-ligand 1 (PD-L1) BUV615, PD-L2 APCR700) were projected onto a two-dimensional plot by UMAP (neighbors=15, minimum distance=0.4, learning rate=1, epochs=200). The dataset was clustered with FlowSOM (distance metric=euclidean) and resulting clusters were overlaid over UMAP plots. Clustered heatmaps showing fluorescence intensity were generated in the OMIQ analysis platform.

### Tumor cell lines and glutamate measurements during melanoma cell culture

We used the BRAF^V600E^-mutant melanoma cell line D4M.3A (from now on called D4M), generated from a Tyr::CreER; Braf^CA^; Pten^lox/lox^ transgenic melanoma mouse model,^25^ and Grm1 expressing Grm1 melanoma cells derived from the tumors of tg(Grm1)EPv mice (generated by A Bosserhoff). All cell lines were cultured at 37°C and 5% CO₂ in cell culture flasks containing suitable cell culture medium (online supplemental table T5—Sigma-Aldrich). Cell lines were on average passaged three to four times in a time span of 7–10 days between thawing of the cells and their use in the experiments. Cells were regularly subjected to *Mycoplasma* testing.

Glutamate levels in the supernatant of D4M and Grm1 cells were determined after 2, 24, 48, 72, 96 and 168 hours of cell culture using the Glutamate-Glo Assay (J7021; Promega, Madison, Wisconsin, USA) according to the manufacturer’s instructions. Protein concentrations were determined by Pierce BCA assay (23225; Thermo Scientific) and samples were normalized to total protein content.

### Viability assay: IncuCyte live cell imaging

12 hours before treatment, 1250 Grm1 and D4M cells were seeded in a 96-well plate (Corning). Before the start of the measurements the live/dead dye DiYO−3 (250 µM—AAT Bioquest, Pleasanton, USA) and the inhibitors telaglenastat (1 µM—MedChemExpress, New York, USA), LY367385 (10, 100, 500 µM—MedChemExpress), L-buthionine-(S,R)-sulfoximine (BSO; 100 µM—MedChemExpress), Imidazole-ketone-erastin (IKE; 1 µM—MedChemExpress), R162 (20 µM—MedChemExpress), or the control dimethyl sulfoxide (Sigma-Aldrich) were added. For the experiments investigating the type of cell death induced by glutamate pathway inhibition, the ferroptosis inhibitor Ferrostatin (4 µM—Sigma-Aldrich), pyroptosis/apoptosis inhibitor zVAD-fmk (20 µM—MedChemExpress) and necroptosis inhibitor GSK'872 (10 µM—MedChemExpress) were additionally included. Images were acquired every 2 hours from three areas per well using a 10 x objective in phase contrast and red fluorescent channel in the IncuCyte S3 Live-Cell Analysis System and analyzed using the IncuCyte 2024B Rev2 software. Phase contrast was used to assess cell confluence, while the red fluorescent channel was employed to quantify cell death by measuring the DiYO−3 signal.

### Intracellular ROS FACS

80.000 Grm1 or D4M cells were seeded in a 48-well plate and cultured overnight at 37°C with 5% CO₂. The cells were treated with the inhibitors LY367385 (10 µM), BSO (100 µM), or IKE (1 µM) for 24 hours at 37°C in 5% CO_2_. Half an hour before dichlorodihydrofluorescein diacetate (DCFDA—Thermo Fisher) staining, the positive control was treated with 250 µM Tert-Butyl hydroperoxide (TBHP—Sigma-Aldrich). Subsequently, cells were detached and incubated in 2’,7’-Dichlorodihydrofluorescein-diacetate /Dulbecco’s Modified Eagle’s Medium 10 µM mix for 10 min at 37°C in 5% CO_2_. All samples were stained with 100 µL of live/dead dye e780.

### Lipid peroxidation assay

Lipid peroxidation was investigated through flow cytometry using BODIPY™ 581/591C11 dye (Sigma). 20.000 Grm1 or D4M cells were seeded in a 48-well plate and cultured overnight at 37°C with 5% CO₂. On the following day, the cells were treated with the inhibitors BSO (100 µM), IKE (1 µM), or RAS-selective lethal 3 (RSL3—Thermo Fisher, 5 µM) for 1 hour at 37°C in 5% CO_2_. Subsequently, cells were detached and stained with live/dead dye e780 for 3 min at room temperature, then incubated with 2 µM BODIPY C11 (581/610) dye for 15 min at 37 °C in PBS. Unincorporated dye was removed by washing with PBS. Oxidized BODIPY-C11 (green emission at 510 nm) was detected.

### Extracellular ATP assay

Cells were seeded in white flat-bottom 96-well plates (Greiner Bio-One, Kremsmünster, Austria) in HEPES-buffered culture medium (PAN-Biotech; Sigma-Aldrich) and allowed to adhere overnight at 37°C. The following day, cells were treated with the inhibitors BSO (100 µM), IKE (1 µM), or Doxorubicin (Merck, Darmstadt, Germany) as a positive control to induce ATP release. Extracellular ATP release was measured using the RealTime-Glo Extracellular ATP Assay (GA5010; Promega) according to the manufacturer’s protocol. Bioluminescence was recorded kinetically in a plate reader at 37°C without CO₂ supply for 24 hours.

### RNA isolation

For real-time quantitative PCR (RT-qPCR), RNA from mouse skin and tumors was isolated using TRIzol Reagent (Life Technologies, Carlsbad, California, USA) according to the manufacturer’s instructions. Frozen samples were disrupted and homogenized on ice in an appropriate volume according to the manufacturers’ recommendations by using a Tissue Ruptor (Omni TH Tissue Homogenizer).

For RNA-sequencing (RNA-seq) analysis, EPv tumor/ear samples were weighed and processed using the RNeasy Mini Kit (Qiagen, Hilden, Germany). Frozen samples were disrupted and homogenized on ice in an appropriate volume of lysis buffer according to the manufacturers’ recommendations by using a Tissue Ruptor (Omni TH Tissue Homogenizer). After lysis, samples were centrifuged through a QIAshredder homogenizer. RNA was isolated according to the manufacturers’ advice by adding an additional DNase I treatment (Qiagen). The RNA elution step was repeated twice in the recommended total volume of 50 µl RNase-free water. RNA amount and quality control were assessed via Nanodrop (Thermo Fisher Scientific) and Qubit (Thermo Fisher Scientific) measurements. RNA integrity was validated via Bioanalyzer (Agilent) before library preparation protocol.

### Real-time quantitative PCR

Genomic DNA was removed from total RNA with the RapidOut DNA removal kit (Thermo Fisher Scientific, Lithuania) and was reverse-transcribed into complementary DNA with random hexamers and SuperScriptR II Reverse Transcriptase (Life Technologies, California, USA) according to the kit’s instructions. PCR analysis was performed on a BioRad CFX96 using Brilliant III Ultra-Fast Master Mix (Agilent Technologies), were selected by Primer Express software (Applied Biosystems, California, USA) and synthesized by Microsynth (Balgach, Switzerland). Primers used for gene expression analysis are summarized in online supplemental table T6.

### RNA sequencing

Lexogen QuantSeq 3’-messenger RNA (mRNA) libraries were prepared at the MultiOmics Sequencing Core Facility (Medical University of Innsbruck) following the manufacturer’s instructions (Lexogen GmbH, Vienna, Austria), quality validated, multiplexed and sequenced with an Illumina NovaSeq sequencer (Azenta, Leipzig, Germany) at 150 bp read length.

### RNA seq data processing and visualization

RNA-seq data was processed with the nf-core RNA-seq pipeline V.3.14.0 (https://nf-co.re/rnaseq/3.14.0) with the “star_salmon” option^26 27^ and the mouse reference genome: GRCm39, M30. Processing encompassed data quality control, read alignment, and gene expression quantification.

Genes with names starting with “Gm” or “Rp” or ending with “rik” were not considered for the downstream analysis. R 4.4.1 was used for downstream analysis.

Analysis and visualization of Gene Ontology (GO) terms associated with differentially-expressed genes was performed using Metascape.^28^ Both groups of genes (upregulated and downregulated, adjusted p value <0.05) were used for GO-derived biological processes, molecular functions and cellular components. The biological terms were grouped together based on their shared genes where the similarity between terms was calculated using kappa statistics.

Heatmaps of count-per-million normalized counts were subjected to z-score across conditions, and plotted with GraphPad Prism V.10.2.3.

### Mouse experimental manipulations

Intratumoral (i.t.) injections into mouse ear tumors were performed under anesthesia in a final volume of 25 µL per ear. Anesthesia was induced with intraperitoneal (i.p.) administration of a mix of phosphate-buffered saline (PBS)-Ketamine-Xylasol (Ratio 1:1:2; aniMedica, Senden-Bösensell, Germany) with a final volume of 100 µl. EPv mice at the transition from TF to TE stage were treated with either PBS, BSO, DC boost, or combination (DC boost+BSO) therapy. DC boost consisted of daily injections of 10 µg Fms-related tyrosine 3 ligand (Flt3L—Celldex Therapeutics, New Jersey, USA) i.p. during the first week of treatment and weekly i.t. injections of polyI:C (25 µg—Sigma-Aldrich) and anti-CD40 (25 µg—produced and provided by Louis Boon). BSO injections consisted of daily i.p. injections of 0.05 mmol/mouse. Mice in the combination group received treatment with DC boost and GLCL inhibitor BSO. Details on reagents/antibodies used for in vivo treatments are summarized in online supplemental table T7.

### Statistical analysis

Sample size was determined based on a power analysis using preliminary data or estimates from similar studies to detect expected effect sizes with sufficient statistical power at a two-sided significance level of 0.05. Due to resource constraints, a simplified power calculation was performed using (https://clincalc.com/stats/samplesize.aspx), focusing on the primary outcome measure.

Statistical analysis was performed using GraphPad Prism V.10.2.3 (GraphPad Software, San Diego, California, USA). To determine whether parametric or non-parametric statistical tests are appropriate, datasets were examined for normality using D’Agostino-Pearson test. For more groups, statistical significance was determined with one-way analysis of variance followed by Tukey’s multiple comparison test (parametric) or Kruskal-Wallis test followed by Dunn’s multiple comparison test (non-parametric). Kaplan-Meier survival curves were compared using the log-rank (Mantel–Cox) test. A p value of <0.05 was considered statistically significant (*), <0.01 very significant (**), <0.001 highly significant (***) and<0.0001 extremely significant (****). Data is presented as mean±SEM.

## Results

### Metabolic reprogramming and glutamate signaling occurs during melanoma progression in the EPv mouse model

Elevated glutamate levels and overexpression of glutamate receptors, including *GRM1*, have been described in several cancer types, including melanoma.^6^,^8^ To determine the clinical relevance of *GRM1* and glutaminolysis-related genes (glutamine uptake, glutaminolysis, and redox stress response) in the survival of patients with melanoma we downloaded and analyzed gene expression data from 470 patients with melanoma (SKCM) (nevi and melanoma) from TCGA database (https://pubmed.ncbi.nlm.nih.gov/24071849/). Based on clinical outcome data, we found that heightened expression of *GRM1* (online supplemental figure S1A), as well as upregulation of an extended panel of genes involved in glutaminolysis (online supplemental figure S1B), was correlated with a significantly decreased overall survival in patients with melanoma. These survival associations highlight the connection of *GRM1* overexpression and glutaminolysis in melanoma progression and underscore the potential clinical benefit of developing therapies targeting *GRM1* overexpression and glutaminolysis specifically in human melanoma.

**Figure 1:**
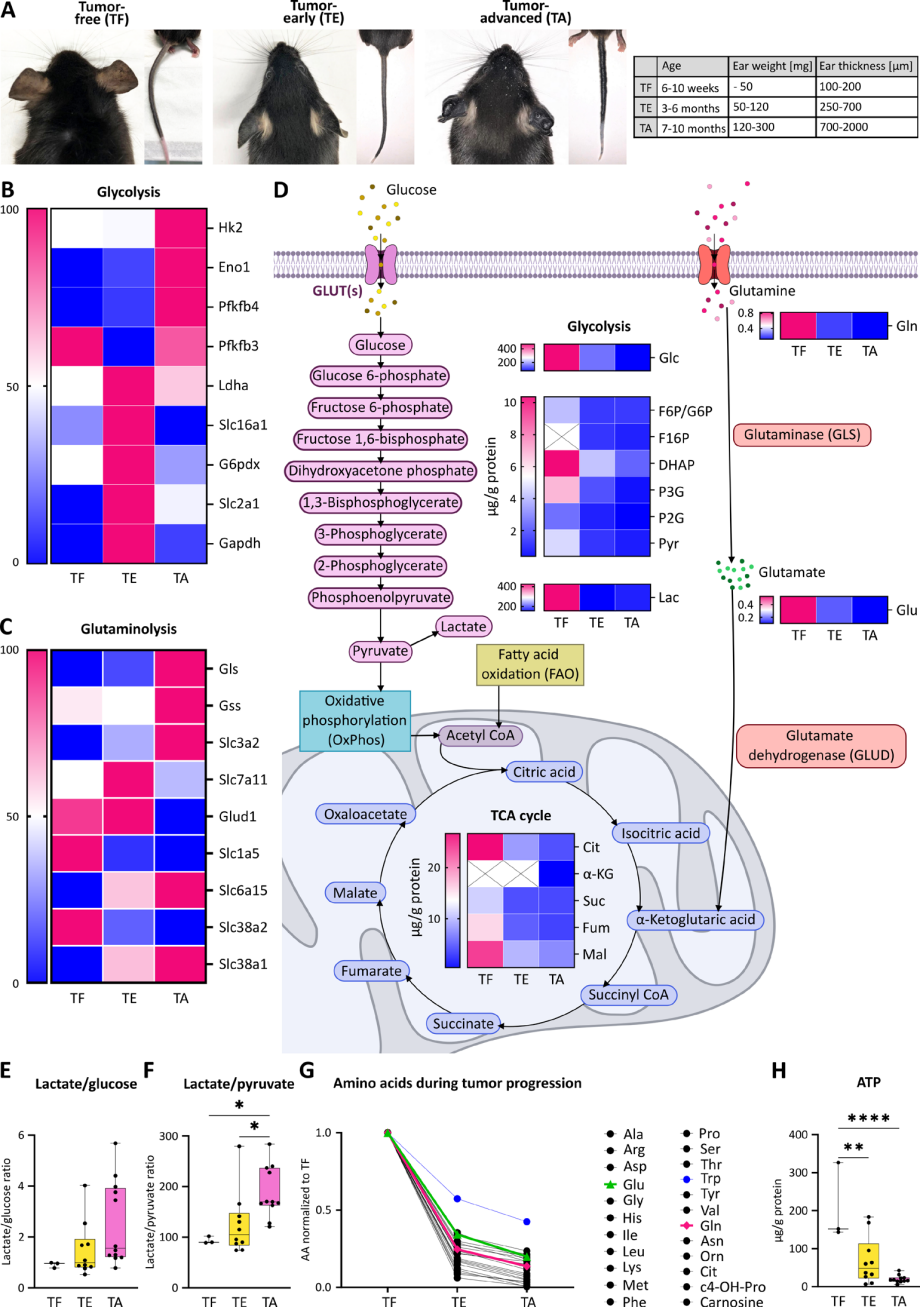
Metabolic reprogramming and glutamate signaling drive melanoma progression in the EPv mouse model. (**A**) Representative pictures of EPv mice at the different tumor stages and table displaying ear thickness and weight measurements for each tumor stage. (**B and C**) RNA-seq analysis was performed with skin/tumor tissue from TF, TE and TA EPv mice. The heatmaps depict normalized and relative RNA-expression (z-score) levels of markers for (**B**) glycolysis and (**C**) glutaminolysis (mean expression n=3). (**D–H**) LC-MS-based metabolomics was performed with lysates of skin/tumor tissue from TF, TE and TA EPv mice. (**D**) Scheme of the key enzymatic steps of glycolysis, TCA cycle, and glutaminolysis in mammalian cells. Total metabolite concentrations for glycolysis, TCA cycle, and glutaminolysis are shown during tumor progression. (**E and F**) Changes in (**E**) lactate to glucose ratio, and (**F**) lactate to pyruvate ratio are shown during tumor progression. (**G**) Amino acid fractions normalized to TF (healthy tissue) are shown during tumor progression. (**H**) Changes in ATP concentration are shown during tumor progression. For (**D–F, H**) results from two independent experiments are shown (n ≥3 mice/group). For (**G**) results from one experiment are shown (n ≥8 mice/group). Statistical significance was determined using mixed-effects model followed by Šídák’s multiple comparisons test, one-way ANOVA followed by Tukey’s multiple comparison test, Kruskal-Wallis test followed by Dunn’s multiple comparison test, or Brown-Forsythe and Welch ANOVA test followed by Dunnett’s T3 multiple comparisons test. Box and whisker plots showing all points min to max. *p<0.05; **p<0.01; ***p<0.001; ****p<0.0001. ANOVA, analysis of variance; LC-MS, liquid chromatography-mass spectrometry; RNA-seq, RNA sequencing; TCA, tricarboxylic acid.

EPv mouse tumors were staged by ear thickness and weight as TF, TE, and TA (see figure 1A for tumor-stage definition). Initial bulk RNA-seq analysis of mouse skin/tumor material displayed predominantly upregulated expression patterns of genes involved in glycolysis and glutaminolysis with tumor growth (figure 1B,C). The GO Biological Process glutamine metabolic processes gene set revealed a more variable expression with an overall trend toward upregulation during tumor progression (online supplemental figure S2A).

Given these findings, we decided to explore Grm1-driven metabolic reprogramming in detail using the autochthonous EPv mouse model. The autochthonous EPv mouse model spontaneously develops melanoma in the skin of the ears, tail, and perianal area due to the ectopic expression of Grm1 under a melanocyte-specific promoter.^9^ A hallmark of metabolic reprogramming in melanoma is increased aerobic glycolysis, also known as the Warburg effect, characterized by increased lactate production in normoxic conditions.^29^ Furthermore, it was previously reported that melanoma cells increase their glutamine uptake and glutaminolysis to produce glutamate via GLS to facilitate upregulated mitochondrial OxPhos.^30^ In this study, we aimed to identify changes in metabolism during tumor progression in the EPv mice.

Metabolite levels during tumor progression in the EPv mice were quantified with targeted LC-MS/MS in ear skin from TF, TE, and TA EPv mice. Overall, metabolites involved in glycolysis, TCA cycle, and amino acid metabolism were reduced during tumor progression (figure 1D, online supplemental figure S2C). To assess the specific contributions of glucose, lactate, and pyruvate within glycolysis, we calculated the lactate-to-glucose and lactate-to-pyruvate ratios (figure 1E,F). The increase in both ratios indicates a switch towards aerobic glycolysis.

Although total glutamine and glutamate concentrations decreased during tumor progression (figure 1D, online supplemental figure S2C), α-ketoglutarate, a key metabolite produced during glutaminolysis, uniquely increased in TA mice (figure 1D). This increase of α-ketoglutarate combined with the overall decrease in other TCA metabolites, suggests that α-ketoglutarate is replenished via glutaminolysis. To further investigate the source of α-ketoglutarate during EPv tumor progression, we analyzed TCA cycle gene expression. TCA cycle genes were generally downregulated, with oxoglutarate dehydrogenase and dihydrolipoamide S-succinyltransferase among the few genes upregulated during tumor progression. In addition, isocitrate dehydrogenase subunits *Idh3b* and *Idh3g* were downregulated while *Idh3a* remained relatively stable, arguing against α-ketoglutarate accumulation via canonical TCA-cycle flux (online supplemental figure S2B). This prompted us to analyze amino acid dynamics. In support of the increased α-ketoglutarate concentration, glutamate was among the amino acids with the lowest reduction during tumor progression, further indicating changes in glutaminolysis (figure 1G). A more comprehensive analysis of all amino acid fractions normalized to TF tissue can be found in online supplemental figure S2D.

The generally reduced metabolite levels were reflected by a significant decrease in ATP in TE and TA melanoma (figure 1H). Tissue ATP levels are maintained through metabolic pathways such as glycolysis, the TCA cycle, and OxPhos.^31^ The significant ATP decrease suggests altered mitochondrial bioenergetics, possibly reflecting hypoxia or mitochondrial dysfunction.

Collectively, our data highlight the metabolic reprogramming during melanoma progression in the EPv mouse model with increased aerobic glycolysis and glutaminolysis, whereas ATP levels decreased, hinting at increased metabolic stress.

### Tumor progression alters the immune infiltrate in the TME of EPv mice

Earlier work from our group demonstrated increased infiltration by MDSC in late-stage EPv tumors. These MDSC could suppress melanoma-specific T cell responses and coincided with an overall loss of DC during tumor development.^21 22^

**Figure 2:**
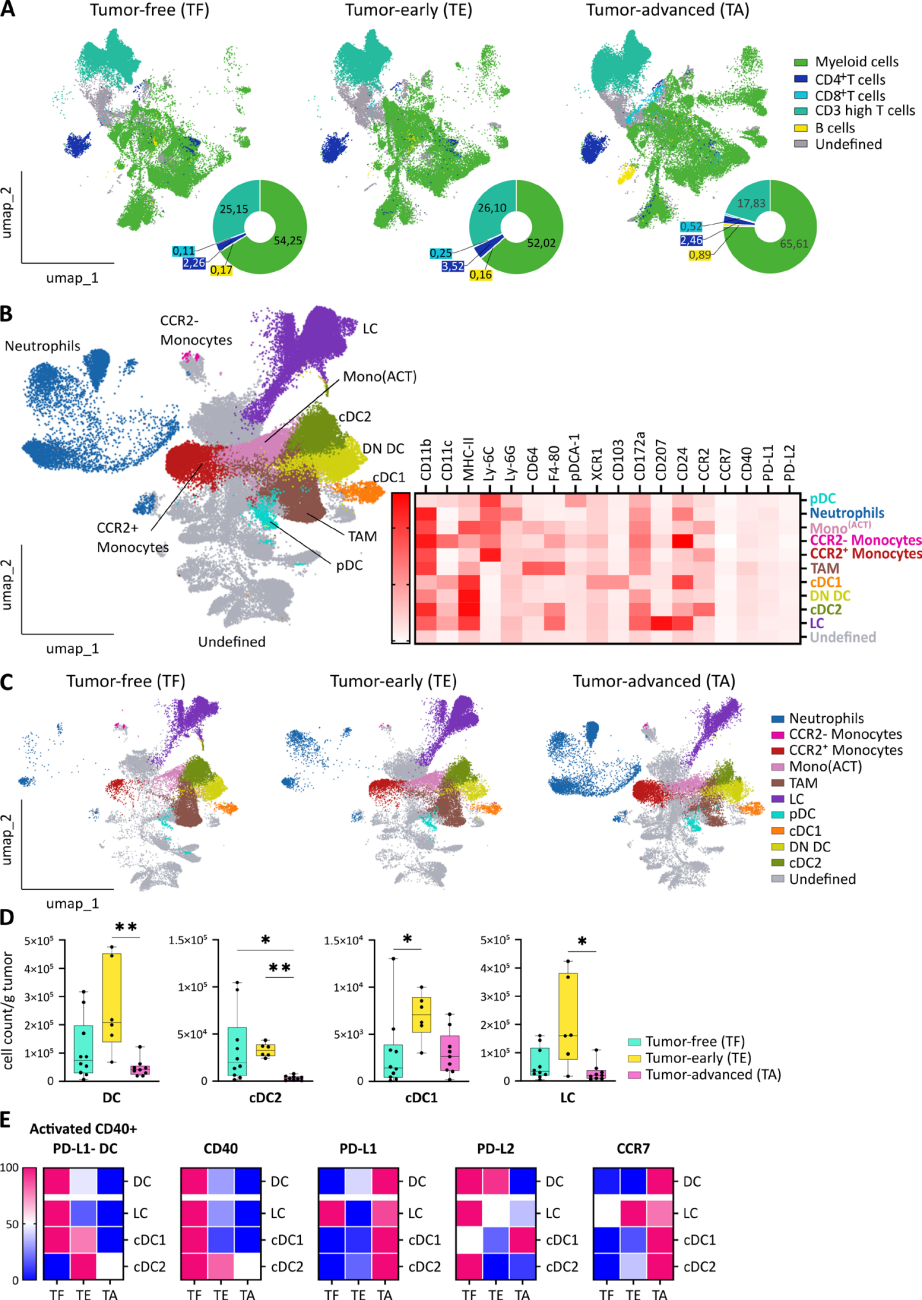
Tumor development impacts the immune infiltrate in the TME of EPv mice. (**A**) Relative abundance of tumor-infiltrating CD4^+^ T cells, CD8^+^ T cells, CD3^high^ T cells, B cells, and myeloid cells from the skin/tumor of TF, TE, and TA EPv mice was determined by flow cytometry. Shown is a UMAP dimensionality reduction of three representative mice per group. (**B**) Flow cytometry data from TF, TE, and TA EPv mouse skin/tumors was concatenated. FlowSOM unsupervised clustering of viable CD45^+^CD3^−^NK1.1^−^CD19^−^ myeloid cells. Left: UMAP of all cells from TF, TE, and TA tissue. Right: heatmap displaying the expression of several myeloid markers on identified clusters across all three groups. (**C**) UMAP of each tumor stage of three representative mice per group displaying the changes in frequencies of the different myeloid cell clusters in skin/tumors. (**D**) Cell numbers of DC subtypes per gram skin/tumors in TF, TE, and TA EPv mice. Cell populations were determined by unsupervised FlowSOM clustering. (**E**) The heatmaps depict the normalized and relative (z-score) DC frequencies and protein expression of CD40, PD-L1, PD-L2, and CCR7. For (**D and E**) results from five independent experiments are shown (n ≥6 mice/group). Statistical significance was determined using one-way analysis of variance followed by Tukey’s multiple comparison test, or Kruskal-Wallis test followed by Dunn’s multiple comparison test. Box and whisker plots showing all points, min to max. *p<0.05; **p<0.01; ***p<0.001; ****p<0.0001. cDC, conventional DC; DC, dendritic cell; DN, double negative; LC, Langerhans cell; MHC, major histocompatibility complex; pDC, plasmacytoid DC; PD-L1, programmed cell death-ligand 1; TAM, tumor-associated macrophages; TME, tumor microenvironment; UMAP, uniform manifold approximation and projection.

To further characterize the immune cell infiltrate and activation status of infiltrating immune cells, we applied a more detailed 26-color flow cytometry panel that we established earlier for mouse skin and lymphatic tissue.^32^ This antibody panel allows a detailed analysis of the different myeloid cell subsets with the simultaneous identification of NK cells, T cells, and B cells. We first focused on the lymphoid cells by identifying the major populations by manual gating (for gating strategy see online supplemental figure S3A,C) and overlaying the UMAP plots (figure 2A).

Given the heterogeneity of the myeloid cell compartment, we decided to perform an unbiased clustering analysis of the flow cytometry data using FlowSOM.^24^ For the analysis, we excluded dead cells, NK, T, and B cells, and used the myeloid cell gate (see online supplemental figure S3B). We identified 11 clusters that were projected on a UMAP space, and all of them were assigned to a specific myeloid subset according to their surface marker expression (figure 2B). We characterized neutrophils, TAM, three monocyte clusters (CCR2^−^ monocytes, CCR2^+^ monocytes, and Mono(ACT)), and four DC clusters (pDC, cDC1, cDC2, and double negative (DN)-DC). Neutrophils were identified by their expression of Ly-6C as well as high levels of Ly-6G and CD11b, and TAM based on the expression of F4/80, and FcγRI/CD64. All monocyte clusters expressed Ly-6C and CD11b, while CCR2^+^ monocytes had higher levels of Ly-6C than CCR2^−^ monocytes. The Mono(ACT) subset expressed the inflammatory chemokine receptor CCR2 in addition to major histocompatibility complex (MHC)-II and CD64. We also identified a cluster that did not express CD11b, CD11c or MHC-II and lacked the expression of monocyte and macrophage markers, that we therefore named “undefined cluster”.

Melanoma development in EPv mice had a strong impact on the myeloid immune cell compartment in the TME, as visualized in the UMAP space with many myeloid clusters shifting in relative frequency (figure 2C). Absolute numbers of myeloid subsets showed that Mono(ACT), CCR2-monocytes, and TAM initially increased during early tumor stages but subsequently decreased in advanced tumor samples (online supplemental figure S4A). This might indicate an initially still functional immune response that becomes suppressed during advanced tumor development. Interestingly, CCR2^+^ monocytes, as well as potentially suppressive neutrophils, were significantly increased in tumor-advanced samples. In line with immune exhaustion, the expression of the activation marker CD40 was decreased on most myeloid cell subtypes, whereas the inhibitory marker PD-L1 was increased during tumor progression (online supplemental figure S4B).^33^ All DC subsets showed an initial increase in their numbers in the TE stage, with a decline to TF-stage numbers in advanced tumors (figure 2D). Activated CD40^+^PD-L1^−^ cells gradually decreased in all DC subsets during tumor development (figure 2E). Correspondingly, CD40 expression was low, while PD-L1 expression was high in DC subsets during tumor progression. Notably, the chemokine receptor CCR7 was elevated on all DC in advanced melanoma lesions of EPv mice.

In summary, early tumors displayed an initially active immune response with increased numbers of pDC, CCR2^−^ monocytes, TAM, and all DC subsets. On tumor progression, immune exhaustion became more prevalent with high numbers of potentially suppressive neutrophils and a lack of activated DC.

### *Grm1*-expressing murine melanoma cells are dependent on the glutathione pathway highlighting potential therapeutic intervention

**Figure 3:**
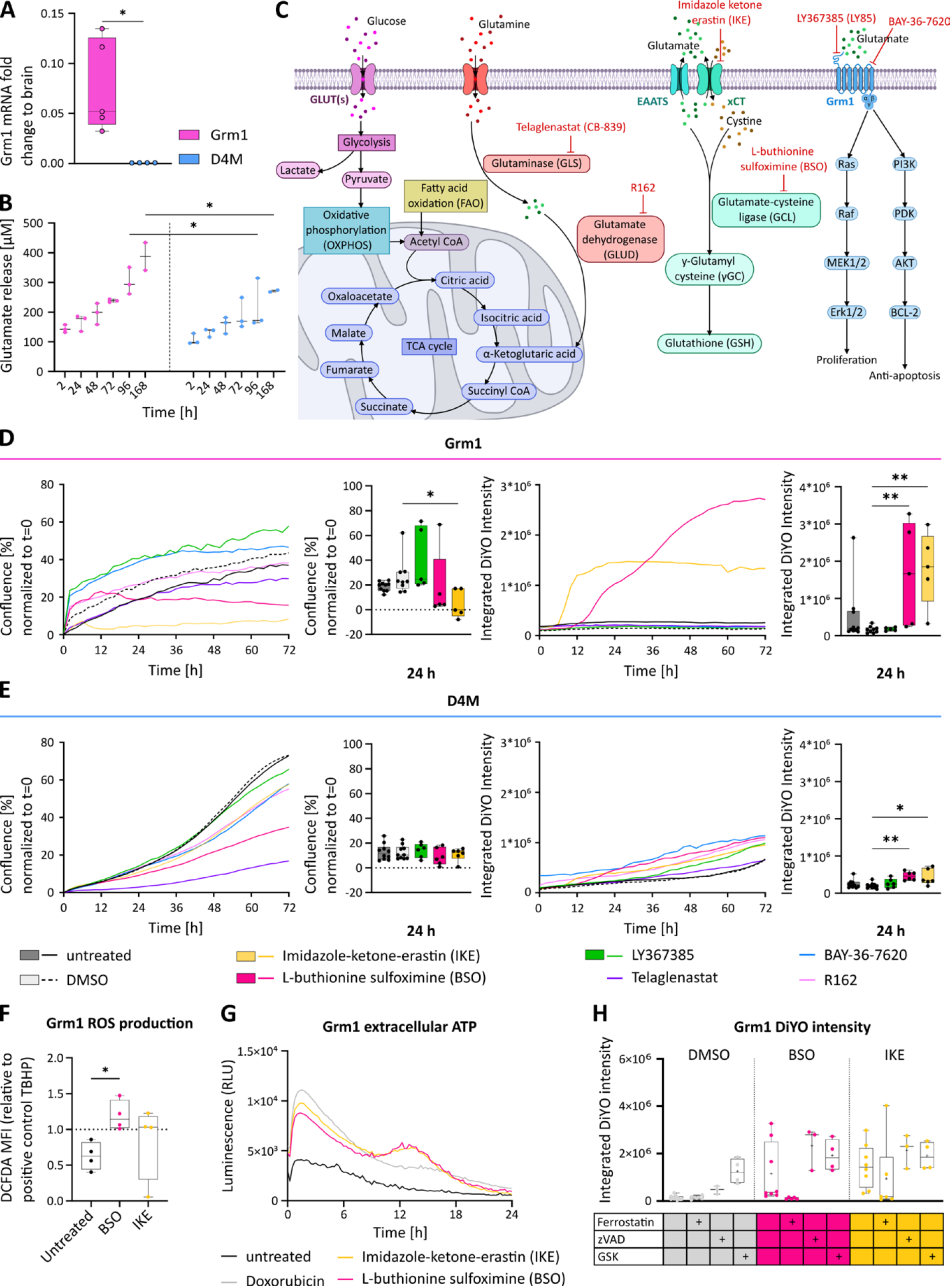
Grm1-expressing murine melanoma cells are dependent on glutamate metabolism, indicating a possible therapeutic vulnerability. (**A**) Quantification of Grm1 mRNA expression in mouse melanoma cell lines Grm1 and D4M using qRT-PCR. Results from three independent experiments (n ≥4). (**B**) Glutamate secretion in µM by Grm1 and D4M melanoma cells (n ≥3). (**C**) Scheme of the main metabolic pathways involving glutamate and glutamine in mammalian cells. Key enzymatic steps are depicted, including glutaminolysis, conversion to α-ketoglutarate via GLUD, as well as entry into the TCA cycle, the glutathione, and Grm1 pathway. Inhibitors targeting specific steps within these pathways are marked in red: Telaglenastat, R162, BSO, IKE, LY367385, and BAY-36-7620. (**D and E**) Cell confluence (normalized to the starting confluence at t=0) and cell death (determined by DiYO intensity) of (**D**) Grm1 and (**E**) D4M cells treated with glutamate pathway inhibitors IKE (1 µM), BSO (100 µM), LY367385 (10 µM), Telaglenastat (1 µM), BAY-36-7620 (50 µM), and R162 (20 µM) compared with controls with medium or 4% DMSO. The 24 hours time point is displayed as whisker plots. Results from five independent experiments (n ≥3). (**F**) Intracellular ROS production in Grm1 cells after 24 hours treatment with 100 µM BSO and 1 µM IKE. Results from four independent experiments (n=4). (**G**) Extracellular ATP release by Grm1 cells treated with 100 µM BSO, 1 µM IKE, or the positive control 5 µM doxorubicin was measured over 24 hours using the RealTime-Glo Extracellular ATP Assay (n=3). (**H**) Grm1 cells were treated with 1 µM IKE, 100 µM BSO, or 4% DMSO (control) together with 4 µM ferroptosis inhibitor Ferrostatin, 20 µM pyroptosis/apoptosis inhibitor zVAD-fmk, or 10 µM necroptosis inhibitor GSK'872. Results from three independent experiments (n ≥3). Statistical significance was determined using mixed-effects model followed by Šídák’s multiple comparisons test, one-way analysis of variance followed by Tukey’s multiple comparison test, or Kruskal-Wallis test followed by Dunn’s multiple comparison test. Box and whisker plots showing all points, min to max. *p<0.05; **p<0.01; ***p<0.001; ****p<0.0001. DCFDA, dichlorodihydrofluorescein diacetate; DMSO, dimethyl sulfoxide; Grm1, metabotropic glutamate receptor 1; MFI, mean fluorescence intensity; mRNA, messenger RNA; qRT-PCR, quantitative real-time PCR; RLU, relative light unit; ROS, reactive oxygen species; TBHP, Tert-Butyl hydroperoxide; TCA, tricarboxylic acid.

Previous work by Shin *et al* showed that *GRM1*-expressing human melanoma cells release elevated levels of glutamate, promoting cell proliferation.^34^ To determine whether similar mechanisms might operate in murine melanoma models, we performed qRT-PCR to quantify *Grm1* mRNA in different murine melanoma cell lines. Of the lines tested, the BRAF^V600E^-mutant melanoma cell line D4M.3A^25^ completely lacked *Grm1* mRNA expression, whereas the Grm1 cell line, which was generated from the EPv melanoma mouse model, expressed *Grm1* (figure 3A). Moreover, we detected excess glutamate release in *Grm1*-expressing melanoma cells (figure 3B).

Glutamine and glutamate metabolism is involved in many cellular signaling pathways, so we tested potential inhibitors to interfere with melanoma cell proliferation (figure 3C). LY367385, a competitive GRM1 antagonist, binds to the same site as the natural ligand glutamate,^35^ while BAY36-7620, a non-competitive Grm1 antagonist, interferes with the transmembrane domain of the receptor.^36^ IKE, a variant of erastin and a known ferroptosis inducer, inhibits the SLC7A11/system xCT to halt the cysteine import and glutamate export of a cell. BSO inhibits GCLC, halting glutathione (GSH) production.^37^ Telaglenastat (CB-839) is a potent oral inhibitor of GLS,^5^ while R162 is a potent small molecule inhibitor of the glutamate dehydrogenase 1.^38^

We used these GRM1 antagonists, glutamine and glutamate pathway inhibitors to examine their impact on the proliferation and induction of cell death in murine melanoma cell lines (Grm1 and D4M) using the Incucyte live cell imaging system for real-time cell viability monitoring. DiYO, a cell-impermeable carbocyanine dimer, is non-fluorescent in the absence of nucleic acid, but exhibits a bright red fluorescence signal on DNA-binding in damaged cells. Cell proliferation by confluence measurement and cell death induction via DiYO fluorescence detection were measured over 72 hours of cell culture. In Grm1 cells, BSO and IKE markedly reduced proliferation and induced cell death within 24 hours (figure 3D, online supplemental figure S5A). LY367385 only affected viability at concentrations 50-fold higher than standard (online supplemental figure S6A). In contrast, BSO and IKE had only a negligible effect on the cell growth of D4M cells and induced modest cell death (figure 3E, online supplemental figure S5B). LY367385 remained ineffective even at higher concentrations in D4M cells (online supplemental figure S6B).

Given that BSO and IKE are linked to ferroptosis through the inhibition of the antiporter xCT and the GSH pathway, which are both key regulators of cellular redox mechanisms to protect cells against lipid peroxidation,^39^ we suspected ferroptosis induction. Therefore, we explored the intracellular ROS production using a fluorescent dye (DCFDA) that can be detected by flow cytometry. Mean fluorescence intensity values for each sample group were normalized to the positive TBHP control (for the gating strategy, we refer to online supplemental figure S6C). Both BSO and IKE increased ROS production in Grm1 cells, while only BSO upregulated ROS production in D4M cells (figure 3F and online supplemental figure S6D). Additionally, we analyzed lipid peroxidation of Grm1 tumor cells after 1 h treatment with the inhibitors BSO and IKE in vitro according to the data published by Wiernicki et al. (2022).^40^ We detected a BODIPY 581/591 C11 fluorescence signal for BSO and IKE similar to the positive control RLS3 (online supplemental figure S6E). This effect could not be detected after BSO or IKE treatment of D4M cells (online supplemental figure S6F)*.* We then quantified ATP release into the cell culture supernatant following treatment with BSO and IKE, compared with the positive control, doxorubicin. Indeed, BSO and IKE induced ATP release comparable to doxorubicin within 24 hours in Grm1 cells but not in D4M cells, consistent with ferroptosis induction (figure 3G, online supplemental figure S6G).^41^

To further study BSO and IKE induced cell death, we examined the impact of the ferroptosis inhibitor ferrostatin-1, the apoptosis inhibitor zVAD-fmk, and the necroptosis inhibitor GSK'872 on the survival of inhibitor-treated cancer cells. We found that treatment with ferrostatin-1 reversed BSO-induced cell death in Grm1 and D4M melanoma cells, strongly suggesting induction of ferroptosis (figure 3H and online supplemental figure S6H). No effect on cell viability could be detected with zVAD-fmk or GSK'872, indicating that BSO can neither induce apoptosis nor necrosis. Ferrostatin-1 partially rescued IKE-induced cell death in Grm1 cells, indicating potential involvement of other cell death pathways (figure 3H). In contrast, neither cell death inhibitor could rescue IKE-treated D4M cells (online supplemental figure S6H).

Altogether, our data outline the therapeutic potential of the inhibitors BSO and IKE targeting the GSH and indirectly glutamate metabolism to interfere with redox balance in melanoma cells. These cell death effects are linked to ferroptosis, as indicated by ROS accumulation, ATP release, and rescue by ferrostatin-1.

### Targeting immune suppression and metabolic reprogramming with a combination of BSO and DC boost therapy controls tumor growth in EPv mice

Our data so far identified two potential mechanisms that could hamper anti-tumor immune responses in the EPv mouse model: the decrease of activated i.t. DC and an altered glutamine and glutamate metabolism.

**Figure 4:**
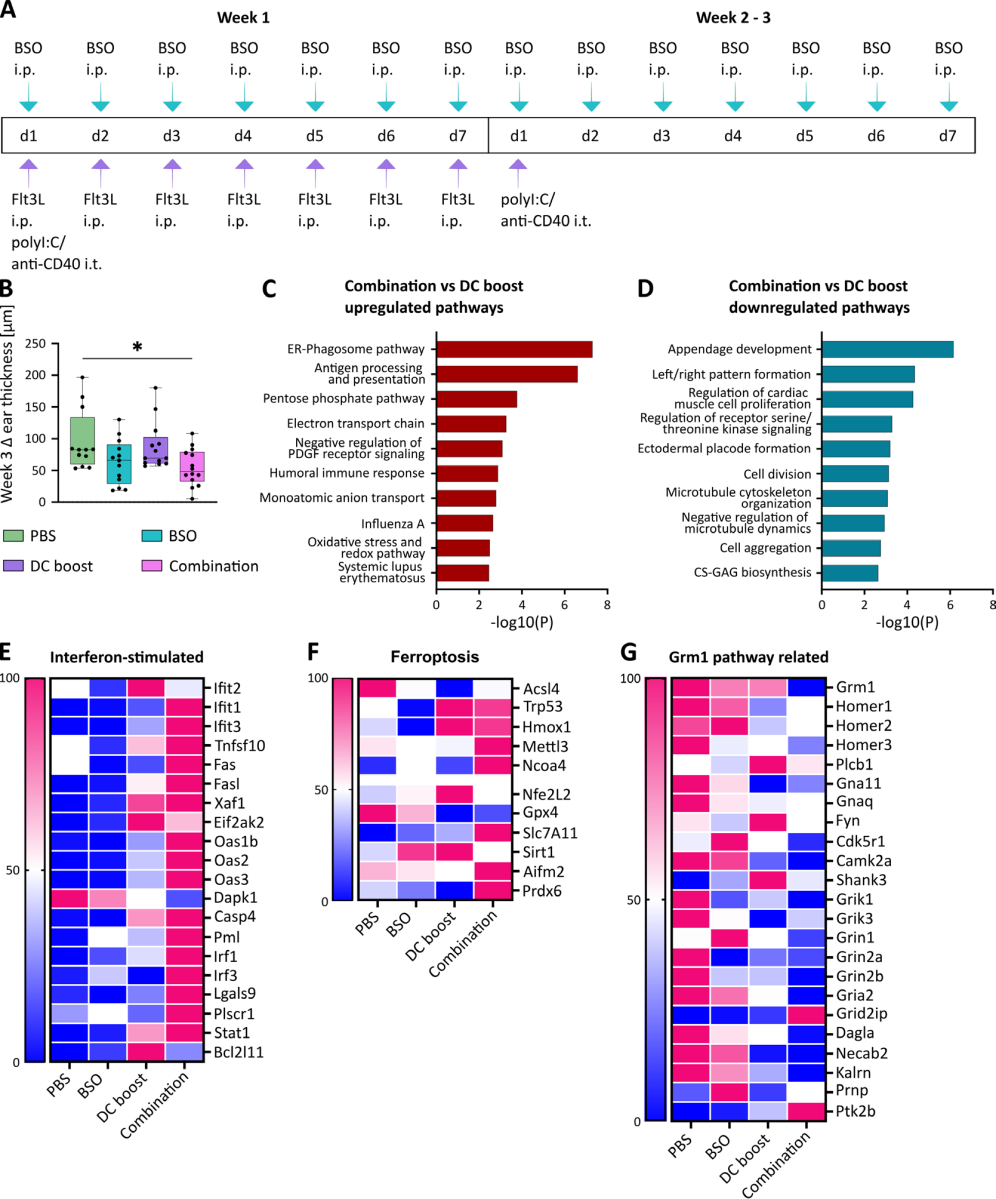
Targeting immune suppression and metabolic reprogramming with a combination of BSO and DC boost therapy controls tumor growth in EPv mice. (**A**) The treatment scheme for DC boost, Gclc inhibition by BSO injection, and combination therapies over 3 weeks is depicted. DC boost consisted of daily injections of 10 µg Flt3L i.p. during the first week of treatment and weekly i.t. injections of polyI:C (25 µg) and anti-CD40 (25 µg). BSO injections were applied daily i.p. in a dose of 0.05 mmol/mouse. Purple arrows indicate DC boost interventions, and turquoise arrows indicate BSO injection. (**B**) EPv mice at the transition from TF to TE stage were treated, and ear thickness changes were measured weekly. Depicted is the ear thickness change reflecting tumor growth at week 3, measured in three independent experiments (n ≥12). (**C–F**) RNA-seq analysis was performed with skin/tumor tissue from PBS, BSO, DC boost, and combination-treated EPv mice (n=5). (**C and D**) ORA was performed on bulk RNA sequencing data, and barplots report the log-scaled p values of (**C**) the most enriched or (**D**) downregulated genes between combination or DC boost monotherapy. (**E–G**) The heatmaps depict normalized and relative expression (z-score) levels of markers for (**E**) ISG, (**F**) ferroptosis driver genes (*Acsl4*, *Trp53*, *Hmox1*, *Mettl3*, *Ncoa4*) and ferroptosis suppressor genes (*Nfe2l2*, *Gpx4*, *Slc7a11*, *Sirt1*, *Aifm2*, *Prdx6*), and (**G**) Grm1 pathway related genes (mean expression n=5). Shown is a UMAP dimensionality reduction of three representative mice per group. Statistical significance was determined using one-way analysis of variance followed by Tukey’s multiple comparison test, or Kruskal-Wallis test followed by Dunn’s multiple comparison test. Box and whisker plots showing all points, min to max. *p<0.05; **p<0.01; ***p<0.001; ****p<0.0001. BSO, L-buthionine-(S,R)-sulfoximine; DC, dendritic cell; Flt3L, Fms-related tyrosine 3 ligand; Gclc, glutamate-cysteine ligase; Grm1, metabotropic glutamate receptor 1; i.p., intraperitoneal; ISG, interferon-stimulated genes; i.t., intratumoral; ORA, over-representation analysis; PBS, phosphate-buffered saline; RNA-seq, RNA sequencing; TE, tumor-early; TF, tumor-free; UMAP, uniform manifold approximation and projection.

We have previously shown that DC boost monotherapy could restore DC numbers and activation status in progressing tumors but was not sufficient to delay tumor growth unless used in combination with ICB.^22^ As an alternative to ICB therapy, we combined the DC boost regimen consisting of systemic administration of Flt3L and i.t. injections with the toll-like receptor-3 ligand polyI:C and an antibody against CD40 with daily i.p. injections of BSO for 3 weeks (treatment scheme shown in figure 4A). The BSO dose (2 mmol/kg) was selected based on a published study.^42^ Mice were treated at the transition from TF to TE stage at 2–4 months of age with an ear thickness<400 µm. At the end of the 3-week-long treatment, the combination therapy significantly inhibited tumor growth when compared with PBS controls as determined by ear thickness measurements (figure 4B). To exclude any toxic effects of the treatment regimen, we determined overall body and liver weight and performed H&E staining on excised liver samples. There was no difference in body or liver weight between the different treatment groups, and liver sections showed no obvious altered structure indicative of liver toxicity (online supplemental figure S7A–C).

To investigate BSO-mediated immunomodulation, we performed RNA-seq analysis of whole tumor/skin tissue after 3 weeks of treatment. For an overall view of the main differences between DC boost monotherapy and combination therapy with BSO, we performed over-representation analysis, which revealed an enrichment in genes reflecting immune response, cellular stress handling, metabolic activity, and cell signaling. These findings indicate a more immunogenic and metabolically active TME in combination-treated mice versus DC boost alone (figure 4C). Combination-treated tumors displayed a downregulation of gene sets linked to development, cell cycle progression, morphogenesis, and structural cellular organization, possibly indicating reduced tumor growth with a shift from growth to protective mechanisms (figure 4D).

IFNs modulate immune responses by regulating genes involved in immune modulation, cell death, and survival.^43^ Given the cell death-inducing effect of BSO in *Grm1*-positive melanoma cells in vitro, we decided to investigate IFN-stimulated genes involved in cell death control and apoptosis. We observed an upregulated expression of several genes with proapoptotic function, including *Fas/CD95, TRAIL/Apo2L/Tnfsf10, Ifit1, Ifit2, Ifit3*, Xaf-1, 2’,5’-oligoadenylate synthetase, *Casp4*, and several others, that in combination, indicate enhanced apoptosis^44 45^ (figure 4E). Furthermore, to define a ferroptosis-related gene signature for both ferroptosis drivers and suppressors, we used FerrDb V3, a manually curated database of ferroptosis regulators and markers.^46^ We observed increased expression of several ferroptosis-driving genes (*Acsl4*, *Trp53*, *Hmox1*, *Mettl3*, *Ncoa4*), whereas ferroptosis suppressors (*Nfe2l2*, *Gpx4*, *Slc7a11*, *Sirt1*, *Aifm2*, *Prdx6*) showed a more heterogeneous pattern indicating a multifaceted cell death response. Notably, the highest-scored suppressor genes, *Nfe2l2* and *Gpx4*, were downregulated in combination-treated mice compared with PBS-treated controls (figure 4F). Combination therapy also decreased expression of GRM1 pathway genes, potentially reflecting reduced tumor cell proliferation, supporting the observed impaired tumor growth (figure 4G).

The combination of DC boost therapy with systemic BSO treatment significantly inhibited tumor growth in the EPv melanoma mouse model without causing toxicity. Combination therapy enhanced the immunogenicity of the TME, induced cellular stress pathways, and altered the metabolic activity of the tissue.

### Combination therapy in EPv mice promotes infiltration of activated DC and T cells

**Figure 5:**
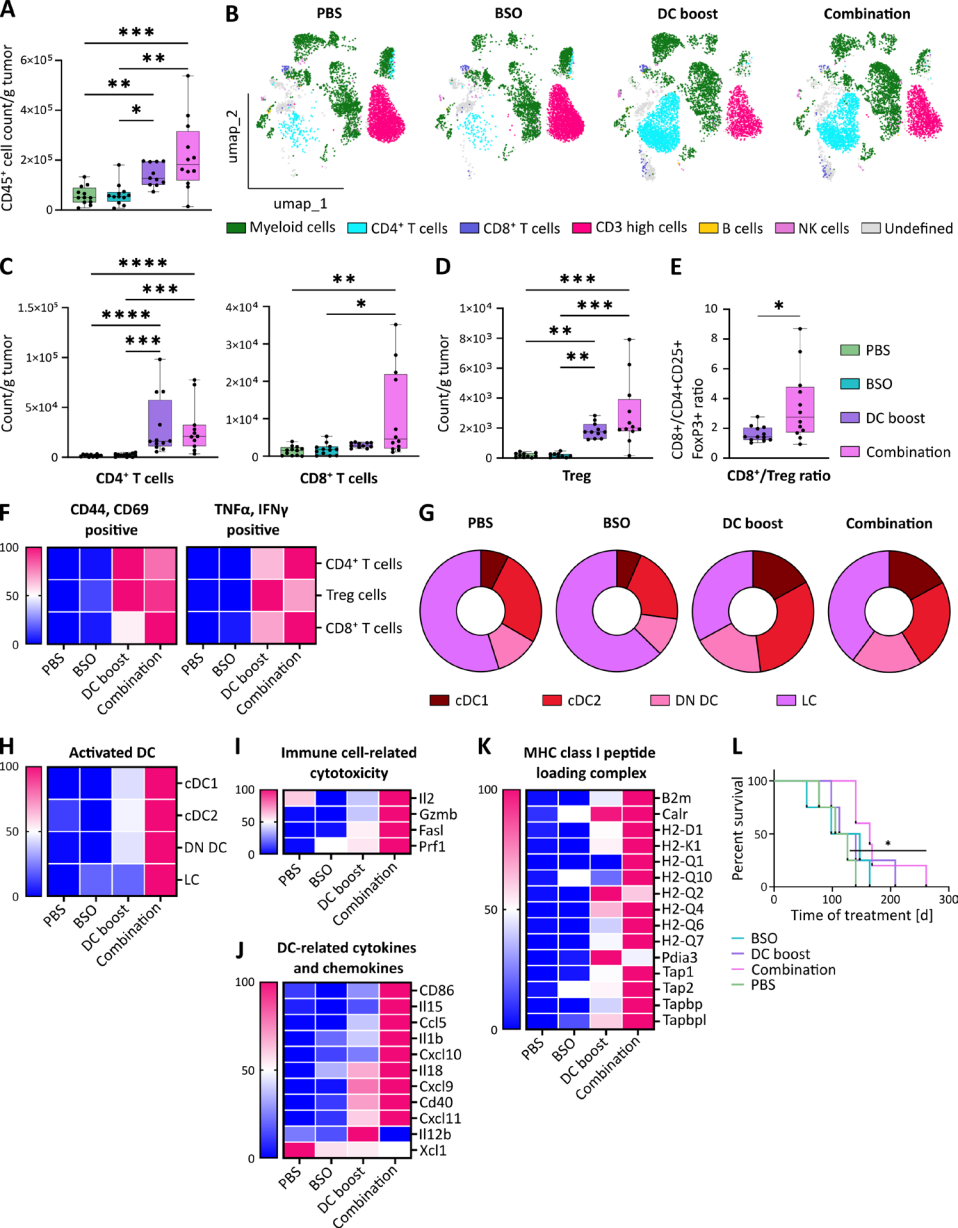
Combination therapy in EPv tumors promotes infiltration of activated DC and T cells. EPv mice at the transition from TF to TE stage were treated with Gclc inhibitor BSO and combination therapies with DC boost over 3 weeks. (**A**) Cell numbers of infiltrating CD45+ immune cells. (**B**) Relative abundance of tumor-infiltrating CD4+ T cells, CD8+ T cells, CD3^high^ T cells, B cells, NK cells, and myeloid cells present in the skin/tumor of PBS, BSO, DC boost, or combination-therapy treated EPv mice was determined by flow cytometry. (**C and D**) Cell numbers of infiltrating (**C**) CD4^+^ and CD8^+^ T cells, and (**D**) regulatory CD4^+^ T cells (Treg). (**E**) Ratio of CD8^+^ T cells to Treg in DC boost and combination-therapy treated EPv mice. (**F**) The heatmaps depict the normalized and relative expression (z-score) frequencies of T cell subsets coexpressing CD44 and CD69, and IFNɣ and TNFα analyzed by flow cytometry at the end of treatment. (**G**) Pie charts displaying the proportions of DC subsets of the overall DC compartment after 3 weeks of treatment. (**H**) The heatmap depicts the normalized and relative expression (z-score) frequencies of CD40 high, PD-L1-DC subsets at the end of treatment. (**I–K**) The heatmaps depict normalized and relative expression levels (z-score) for genes (**I**) important for immune cell-related cytotoxicity, (**J**) DC-related cytokines and chemokines, and (**K**) involved in MHC class I peptide loading complex. For (**A–H**) results from three independent experiments are shown (n ≥12 mice/group). For (**I–K**) n=5 mice/group. (**L**) EPV mice were left without treatment until they reached Animal Care and Use Committee tumor burden limits and overall survival displayed as Kaplan-Meier curve is shown (n ≥4/group). Statistical significance was determined using one-way analysis of variance followed by Tukey’s multiple comparison test, or Kruskal-Wallis test followed by Dunn’s multiple comparison test. Box and whisker plots showing all points, min to max. Kaplan-Meier survival curves were compared using the log-rank (Mantel-Cox) test. *p<0.05; **p<0.01; ***p<0.001; ****p<0.0001. BSO, L-buthionine-(S,R)-sulfoximine; cDC, conventional DC; DC, dendritic cell; DN, double negative; Gclc, glutamate-cysteine ligase; IFNɣ, interferon-gamma; LC, Langerhans cell; MHC, major histocompatibility complex; NK, natural killer; PBS, phosphate-buffered saline; PD-L1, programmed cell death-ligand 1; TE, tumor-early; TF, tumor-free; TNFα, tumor necrosis factor-alpha; Treg, regulatory T cell; UMAP, uniform manifold approximation and projection.

To gain further insights into the immune cell infiltrate and the activation status of infiltrating immune cells, we used our 26-color flow cytometry panel to characterize the immune cell subtypes in treated tumors (gating strategy see online supplemental figure S3). In addition, we applied a 13-antibody panel allowing detailed analysis of T cell subsets (gating strategy see online supplemental figure S8A,B). Analysis revealed more infiltrating CD45^+^ immune cells in response to DC boost monotherapy, with further increase in the combination therapy group (figure 5A). For a more detailed insight into the lymphoid and myeloid cell compartments, we identified the major populations by manual gating (gating strategy see online supplemental figure S3) and overlaid them on UMAP plots (figure 5B).

We observed a recruitment of CD4^+^ and CD8^+^ T cells to the tumor tissue (figure 5C) in the DC boost and combination treatment groups, alongside a recruitment of CD4^+^ regulatory T cells (Tregs) (figure 5D). The ratio of CD8^+^ T cells/Tregs was significantly increased in the combination group compared with the DC boost group, indicating a dominance of potential cytotoxic CD8^+^ T cells over regulatory, immunosuppressive cells (figure 5E). To assess the functionality of recruited T cells post-treatment, we measured expression of activation markers (CD44, CD69) and cytokine production (IFNγ, TNFα). Combination therapy increased the frequency of activated CD44^+^CD69^+^CD8^+^ T cells. At the same time, an increase in IFNγ and TNFα double-producing CD4^+^ and CD8^+^ T cells was observed mainly for the combination-treatment (figure 5F). These results demonstrate that combination therapy stimulated infiltration of activated cytokine-producing CD4^+^ and CD8^+^ T cells into the TME of EPv tumor-bearing mice.

In line with these results, DC boost and combination therapy caused a shift in DC subsets towards cDC1 and DN DC in the TME (figure 5G). Combination therapy significantly increased recruitment of activated CD40^+^PD-L1^−^ DC subsets (cDC1, cDC2, DN DC, and LC) into the tumor, which likely contributed to higher immunogenicity of the TME (figure 5H).

In support of our findings, RNA-seq analysis revealed upregulation of genes involved in T cell function, including those associated with immune cell cytotoxicity (*Prf1, Gzmb, Fasl*) and T cell activation (*Il2*) (figure 5I). Accordingly, markers for DC recruitment and DC-T cell interaction were strongly induced in combination-treated tumors (figure 5J). The chemokine *Ccl5*, for example, mediates cDC1 infiltration into tumors,^47^ whereas *Cxcl9*, *Cxcl10*, and *Cxcl11*, partially through *Il15*, are important for DC-T cell interaction by recruiting CXCR3+ effector T cells.^48^ Moreover, the DC-expressed costimulatory molecules *Cd40* and *Cd86*, essential for T cell stimulation, were strongly upregulated after combination treatment (figure 5J).

In accordance with the higher immunogenicity of the TME, MHC class I peptide loading complex-related genes were broadly upregulated in combination therapy-treated melanoma (figure 5K). This includes augmented expression of heavy chains (H2 family), β2-microglobulin (*B2m*), chaperones (*Calr, Pdia3*), peptide transporters (*Tap1, Tap2*), and peptide editors (*Tapbp, Tapbpl*). Collectively, these changes suggest improved assembly, quality control, and surface display of peptide-MHC class I complexes, promoting robust CD8^+^ T cell recognition and immune surveillance.

At the end of the 3-week treatment study, a random subset of male and female mice was selected to study their long-term survival in the absence of any further treatment. The survival study was performed until the mice reached Animal Care and Use Committee tumor burden limits. Overall, the combination-treated arm showed the highest proportion of mice surviving despite cessation of therapy, suggesting a potential long-term effect by metabolic remodeling and immunotherapy at the same time (figure 5L).

In retrospect, our results reveal a switch in immunogenicity of the TME in response to the combination therapy consisting of an inhibitor of GSH metabolism and DC boost therapy. The infiltration of activated DC and T cells most likely contributes to the inhibition of tumor growth.

## Discussion

The findings from our study demonstrate that metabolic reprogramming of glutamine and glutamate signaling is a key driver of melanoma progression in the transgenic EPv tumor mouse model. The analysis of the immune cell infiltrate revealed expanded populations of pDC, CCR2^−^ monocytes, TAM, and various DC subsets during early tumor stages, followed by an immunologically exhausted status during tumor progression marked by declining numbers of activated DC, higher expression of negative immune checkpoints, and a rise in immunoregulatory cell populations. Notably, EPv melanoma cells were sensitive to inhibition of GSH metabolism and subsequent glutamate metabolism and redox balance, with BSO and IKE inducing cell death, ROS accumulation, increased lipid peroxidation, and ATP release, an effect reversible by the ferroptosis inhibitor ferrostatin-1. In vivo, the combination of BSO and DC boost therapy increased DC and CD8^+^ T cell infiltration and activation, delayed tumor growth, and extended overall survival of tumor-bearing mice, highlighting the therapeutic potential of strategies targeting both metabolic reprogramming and immune cell recruitment.

Tumor cells adapt to the hypoxic, nutrient-deprived TME by upregulating aerobic glycolysis (Warburg effect) and glutamine metabolism. These adaptations are crucial to maintain the rapid proliferation and subsequent biosynthetic and energy needs.^30^ Such metabolic changes have previously been studied in murine and human melanoma, though never with a focus on *Grm1/GRM1* expression.^6 7^ Glutamate receptor overexpression, especially *Grm1*, is increasingly implicated in melanoma pathogenesis through MAPK and PI3K/AKT signaling cascades, promoting proliferation, survival, and angiogenesis.^10^ Overexpression of glutamate receptors and elevated glutamate levels have been described in several cancer types, including melanoma, breast, prostate, and lung cancer.^7^ However, metabolic competition and nutrient restriction in the TME not only impact the tumor cells but can also affect the immune infiltrate in tumors and subsequently tumor immunity.^13^ Our results demonstrate that glutamine and glutamate concentrations decrease in the progressing tumor, which may contribute to the suppressed DC activation and antigen presentation observed in advanced EPv tumors. In line with our observations, glutamine has been reported to be one of the most important amino acids for DC development and function, operating as an intercellular metabolic checkpoint that dictates tumor-cDC1 crosstalk and facilitates cDC1 function. Dietary glutamine supplementation in preclinical tumor models, such as MC38 and B16-OVA, can suppress tumor progression.^49^

Another important factor contributing to immunosuppression in the TME of melanoma is the accumulation of ROS, generated as a byproduct of melanin synthesis.^50^ Besides production by melanoma cells, it has been reported that MDSC and TAM in the hypoxic TME upregulate glycolysis and lipid metabolism, generate excess ROS, and promote immunosuppression through checkpoint expression (PD-L1/PD-L2).^51^ In the EPv mouse model, monocytes, TAMs, and neutrophils exhibited increased PD-L1 and PD-L2 expression, consistent with a suppressive phenotype. The interplay between metabolic stress, ROS production, and immune cell exhaustion fosters a cold tumor environment, resistant to immune-mediated tumor cell clearance. At the same time, the excessive production of ROS and accumulation of iron-associated lipid peroxides can lead to a caspase-independent form of regulated cell death called ferroptosis. GSH is a low-molecular-weight peptide that maintains cellular redox balance by scavenging free radicals and altered GSH metabolism has been linked to drug resistance in melanoma.^52^ Building on these findings, we investigated the vulnerability of *Grm1*-expressing melanoma cells to drugs targeting metabolic and redox pathways.

Indeed, the drug BSO, known to deplete intracellular GSH by inhibiting the activity of the rate-limiting enzyme GCLC,^50^ induced cell death through ROS accumulation. As previously reported, BSO is directly cytotoxic to melanoma cell lines^53^ and increased survival in B16 melanoma-bearing mice.^42^ These effects were particularly pronounced in *Grm1*-positive cells derived from EPv mice, supporting the clinical relevance of targeting glutamatergic signaling in melanoma. We identified the underlying mechanism of cell death as ferroptosis, as we could rescue cells by the addition of ferrostatin-1.

Recently published studies by Wiernicki *et al*^40^ and Liu *et al*^54^ reported inhibitory effects of ferroptosis on DC function. In contrast, we did not observe such negative effects on DC following in vivo treatment with BSO or the combination approach. Instead, our findings appear more consistent with those of Efimova *et al*, who demonstrated that ferroptotic cancer cells can promote efficient antitumor immunity.^55^ Differences between our results and these earlier reports are likely due to variations in experimental design. In our study, tumor-bearing mice were systemically treated with BSO, whereas Wiernicki *et al* and Liu *et al* either injected ferroptotic tumor cells or used in vitro models of ferroptotic cells. Consequently, the level of ferroptosis induced in tumor cells in their settings is likely higher and more acute than in our systemic BSO treatment regimen. Moreover, the tumor models employed, including MCA205 fibrosarcoma cells and BM1 mouse embryonic fibroblasts, are not directly comparable to our *Grm1*-overexpressing mouse tumor model.

In our EPv mouse model, we were able to evaluate the long-term efficacy of compounds on tumor growth and overall survival with a treatment period of 3 weeks and observation for up to 8 months. BSO monotherapy had no effect on tumor progression and underscores the necessity of combination therapy with effective immune engagement for durable tumor control, a concept reflected in recent translational studies that link metabolic rewiring with enhanced immunotherapy responses.^56^

BSO has previously been investigated in combination with alkylating agents, where it enhances DNA damage and the cytotoxic effects of these drugs, helping to overcome therapy resistance in melanoma.^50^ However, these agents are no longer considered standard clinical practice for melanoma. Immunotherapy is now recommended as first-line systemic therapy for patients with melanoma, regardless of BRAF mutational status, while BRAF and MEK inhibitor combinations are reserved for patients resistant to ICB treatment.^57^ Unfortunately, there is still a significant portion of patients with melanoma unresponsive to ICB therapy. Indeed, we showed previously that monotherapy with anti-PD-L1 mAb was not sufficient to delay tumor growth in the EPv mouse model, while in combination with DC boost therapy, tumor progression could be delayed.^22^ Therefore, we tested the potential value of combining systemic BSO treatment with DC boost therapy and observed impaired tumor growth accompanied by a robust antitumor response with DC and T cell infiltration into the TME. When we characterized the immune infiltrate in more detail, we detected higher frequencies of activated CD40^+^PD-L1^−^ cells in all DC subtypes, as well as activated CD4^+^ and CD8^+^ T cells producing TNF-α and IFNγ. This switch to a more immunogenic TME was reflected in the upregulation of genes associated with T cell cytotoxicity, DC-related cytokines and chemokines, and the antigen presentation machinery. Even more striking was the long-lasting effect of the 3-week-long combination therapy on the overall survival of EPv mice in an observation period of 7 months, suggesting a potential long-term effect of metabolic remodeling and immunotherapy at the same time.

This integration of metabolic and immune remodeling suggests that simultaneously addressing tumor-intrinsic metabolic vulnerabilities and the restoration of effective immune surveillance is a promising emerging therapeutic strategy. Our work supports clinical and preclinical data showing that metabolic targeting can enhance the efficacy of immunotherapy, probably also in settings of resistance.^58^ The approach of combining BSO or similar agents with DC boost or alternative immunotherapies such as ICB highlights a shift toward multitargeted treatment regimens, tailored to the metabolic and immunologic features of tumor entities.

Taken together, our findings position metabolic reprogramming and immune suppression as intimately linked drivers of melanoma progression. By dissecting and targeting these processes in tandem, future therapies may achieve deeper and more sustained responses in patients with melanoma, particularly those bearing glutamate pathway-active or immunologically cold tumors.

## Supplementary material

10.1136/jitc-2026-014808 online supplemental file 1

## Data Availability

Data are available upon reasonable request. All data relevant to the study are included in the article or uploaded as supplementary information.
