# Supplementary material for: Metabolic inhibition of glutamate-cysteine ligase increases dendritic cell-mediated antitumor immunity in melanoma
Source: J Immunother Cancer. 2026 Jun 30;14(6):e014808. doi: 10.1136/jitc-2026-014808 (PMC13331211; doi:10.1136/jitc-2026-014808)
Supplement: online supplemental file 1 [file jitc-14-6-s001.pdf]

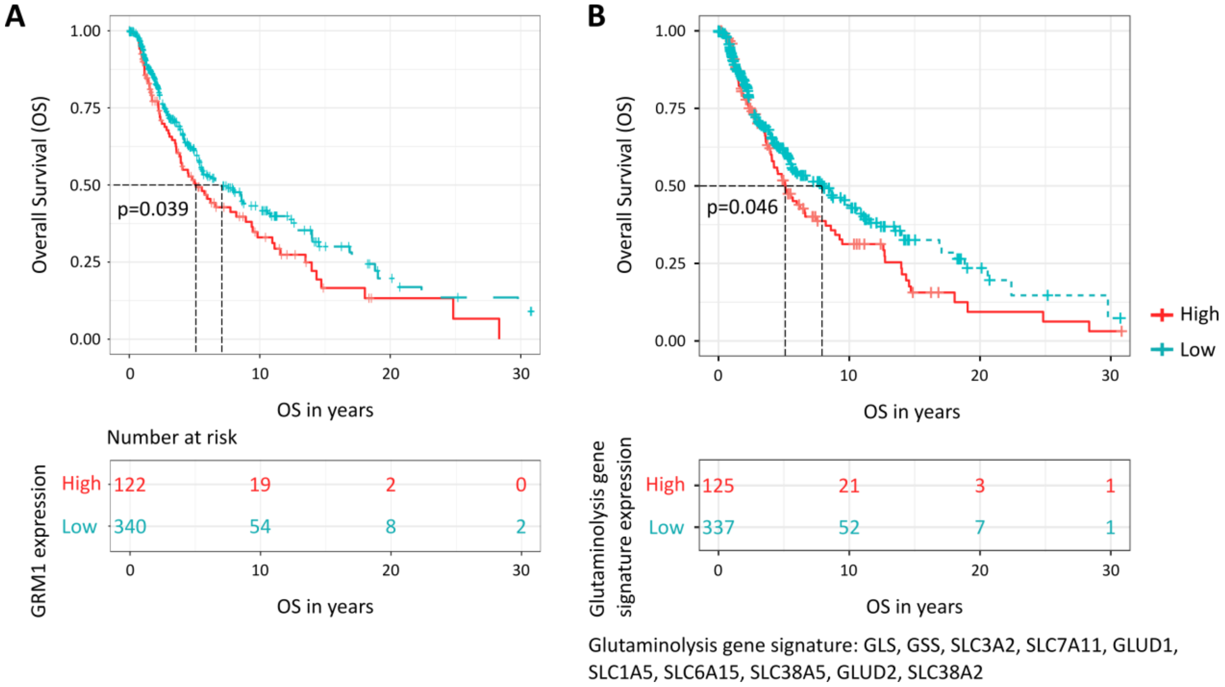


**Supplementary Figure S1. Grm1 expression and genes involved in glutaminolysis impact melanoma patient survival.** Kaplan-Meier curves of overall survival for melanoma patients with high and low expression of **(A)** *GRM1*, and **(B)** glutaminolysis genes (*GLS, GSS, SLC3A2, SLC7A11, GLUD1, SLC1A5, SLC6A15, SLC38A5, GLUD2, and SLC38A2*). P-values were calculated using the log-rank test. *$p < 0.05$; **$p < 0.01$; ***$p < 0.001$; ****$p < 0.0001$.

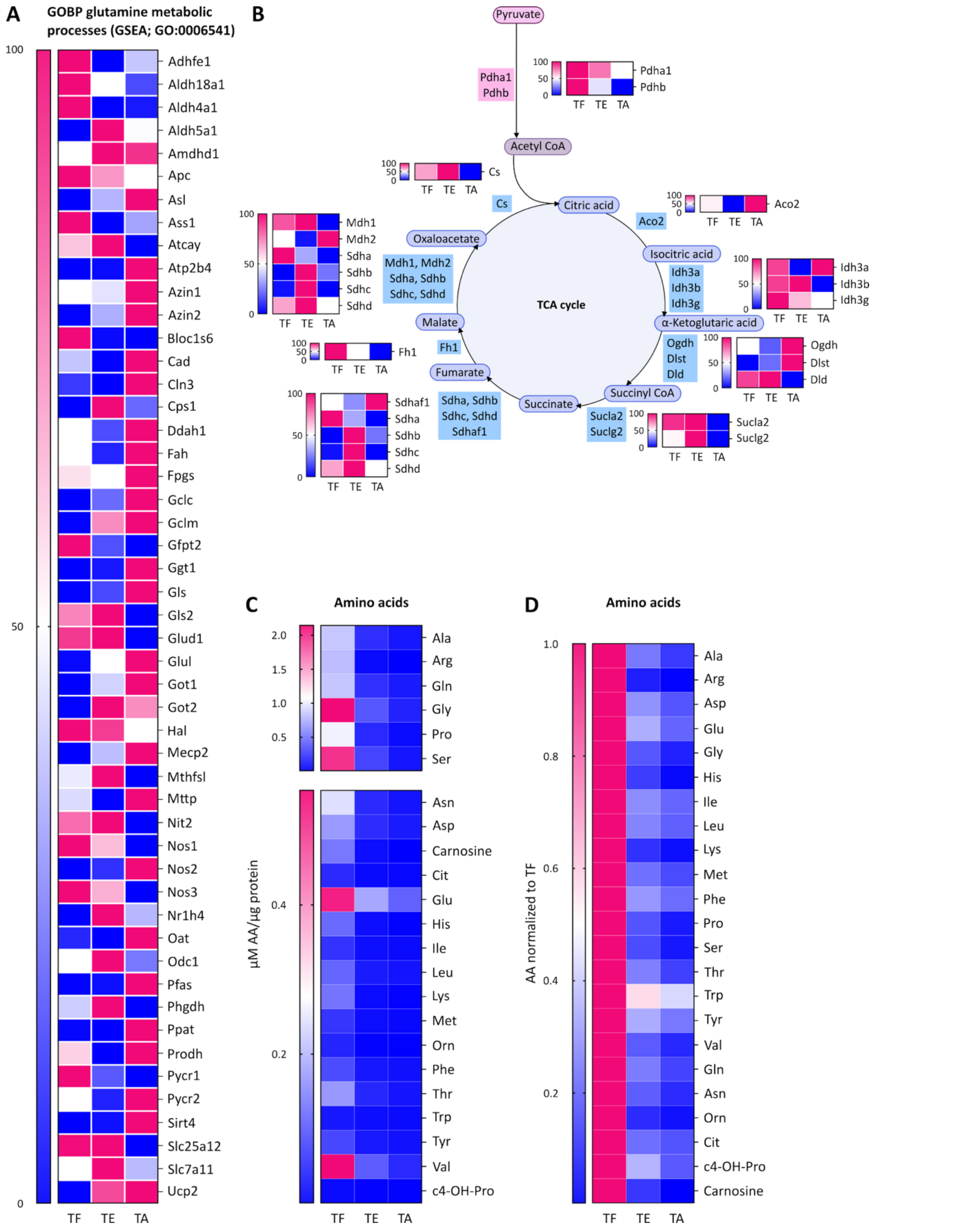


**Supplementary Figure S2. Overview of metabolic pathways involving glutamine and-metabolic alterations in Epv tumors. (A and B)** RNA-seq analysis was performed with skin/tumor tissue from TF, TE and TA EPv mice (mean expression n=3). The heatmaps depict normalized and relative RNA-expression (z-score) levels of markers for **(A)** GOBP glutamine metabolic processes (GSEA; GO:0006541). **(B)** Scheme of the key enzymatic steps of TCA cycle-associated genes. **(C and D)** LC-MS/MS-based metabolomics was performed with lysates of skin/tumor tissue from TF, TE and TA EPv mice. **(C)** Total metabolite concentrations for amino acids are shown during tumor progression. **(D)** Metabolite fractions normalized to TF (healthy tissue) for amino acids are shown during tumor progression. For **(C and D)** results from one experiment are shown (n ≥ 8 mice/group).

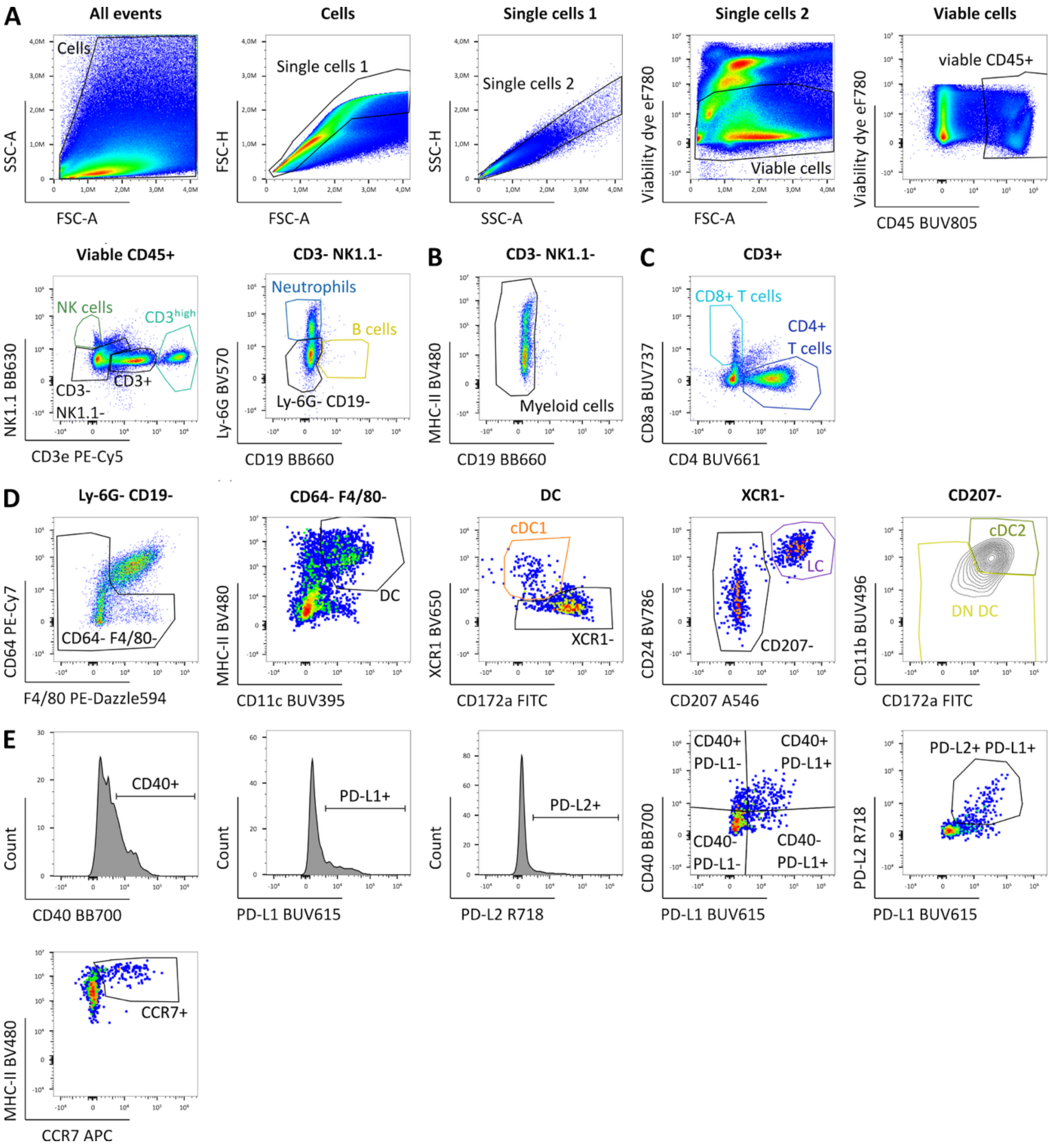


**Supplementary Figure S3. Gating strategy for myeloid cells in mouse ear and tumor ear skin.** Ear skin was enzymatically digested to obtain single-cell suspensions. **(A)** Gating strategy for viable CD45$^+$ cells after exclusion of cellular debris, doublets, and dead cells. CD3$^+$, CD3$^{high}$, NK1.1$^+$ NK cells, CD19$^+$ B cells, Ly-6G$^+$ neutrophils were identified. **(B)** Viable CD45$^+$CD3$^-$NK1.1$^-$CD19$^-$ myeloid cells were gated and used for subsequent FlowSOM unsupervised clustering analysis (shown in Figure 2). **(C)** CD3$^+$CD4$^+$ and CD3$^+$CD8$^+$ T cells were identified. **(D)** The Ly-6G$^-$CD19$^-$ and CD64$^-$F4/80$^-$ population contains CD11c$^+$MHC-II$^+$ DC, which were subdivided into XCR1$^+$ cDC1, CD172a$^+$CD207$^+$CD24$^+$ LC, CD11b$^+$CD172a$^+$ cDC2, and CD11b$^-$ CD172a$^-$ DN DC. **(E)** Expression of CD40, PD-L1, and PD-L2 on DC subsets, Mono(Act), CCR2$^+$ monocytes, CCR2$^-$ monocytes, TAM, and neutrophils was identified, as well as the CCR7 expression on DC subsets. One representative example is shown.

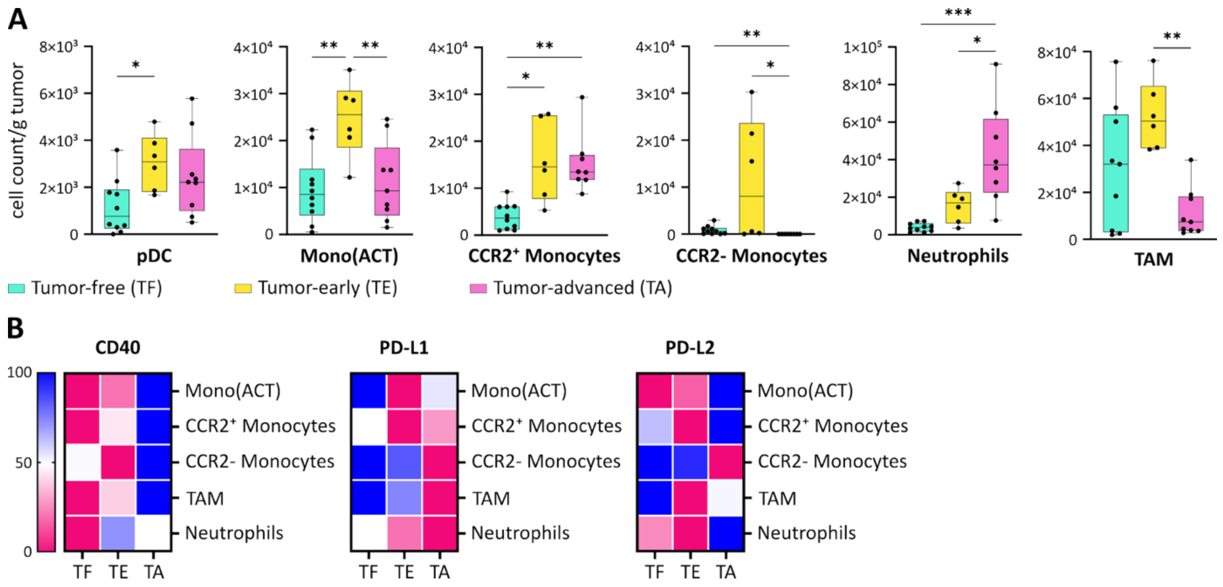


**Supplementary Figure S4. Tumor development alters the immune infiltrate in the TME of Epv mice. (A)** Cell numbers of infiltrating immune cells per gram skin/tumors in TF, TE, and TA EPv mice as determined by unsupervised FlowSOM clustering of myleoid cells pregated according to Supplementary Figure S3B. **(B)** The heatmaps depict the normalized and relative expression (z-score) frequencies of CD40, PD-L1, and PD-L2 expressing Mono(Act), CCR2$^+$ monocytes, CCR2$^-$ monocytes, TAM, and neutrophils. For **(A and B)** results from five independent experiments are shown (n ≥ 6 mice/group). Statistical significance was determined using one-way analysis of variance, followed by Tukey's multiple comparison test, or Kruskal-Wallis test, followed by Dunn's multiple comparison test. Box and whisker plots showing all points, min to max. *p < 0.05; **p < 0.01; ***p < 0.001; ****p < 0.0001.

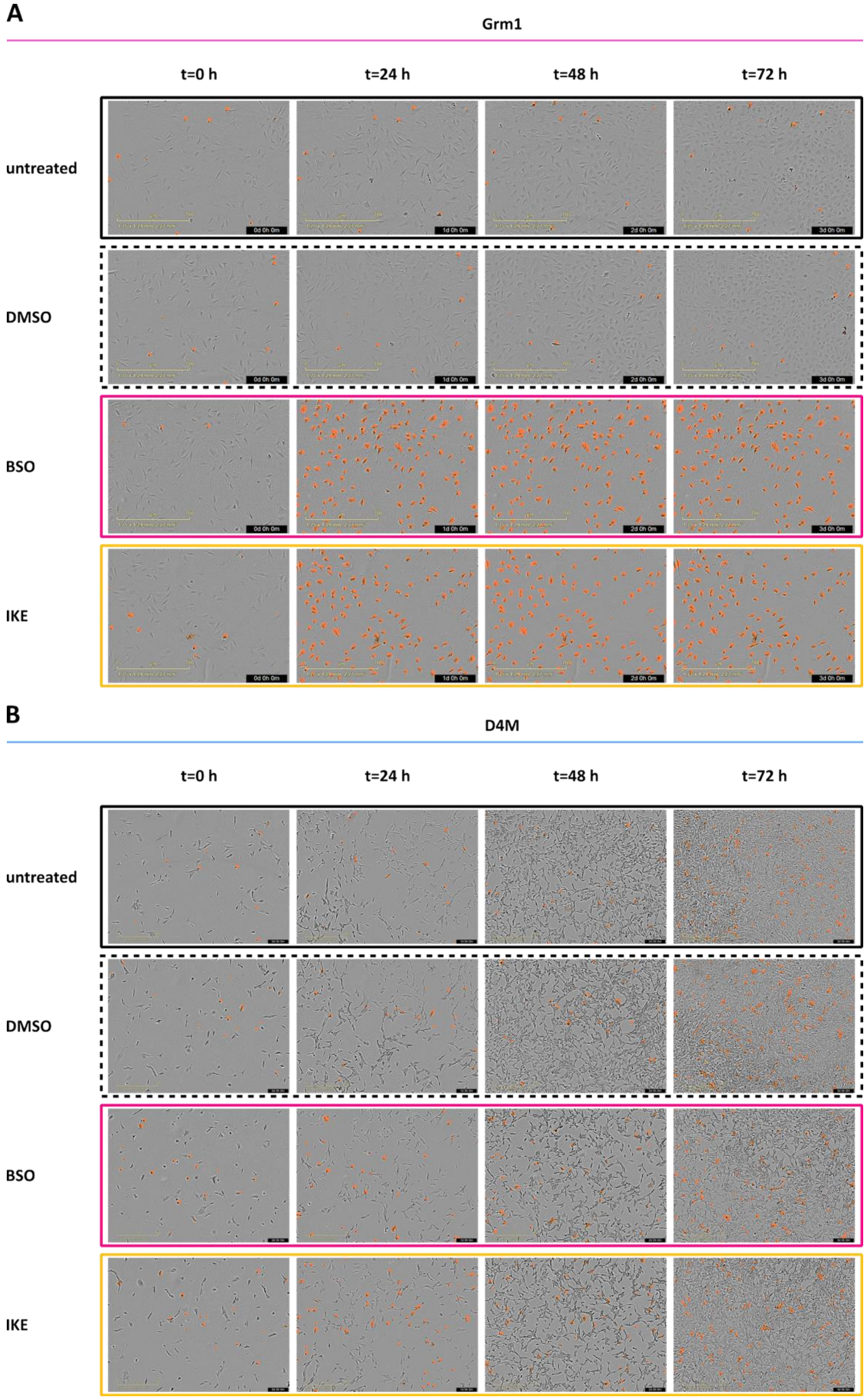


**Supplementary Figure S5. BSO and IKE treatment induced cell death in Grm1 cells.** Evaluating the cytotoxic effects of glutamate pathway inhibitors on the melanoma cell lines Grm1 and D4M. **(A and B)** Representative images of **(A)** Grm1 and **(B)** D4M cells were selected at 4 different time points for each treatment. Red fluorescence (DiYO) indicates dying or dead cells. Size bar displays 700 µm.

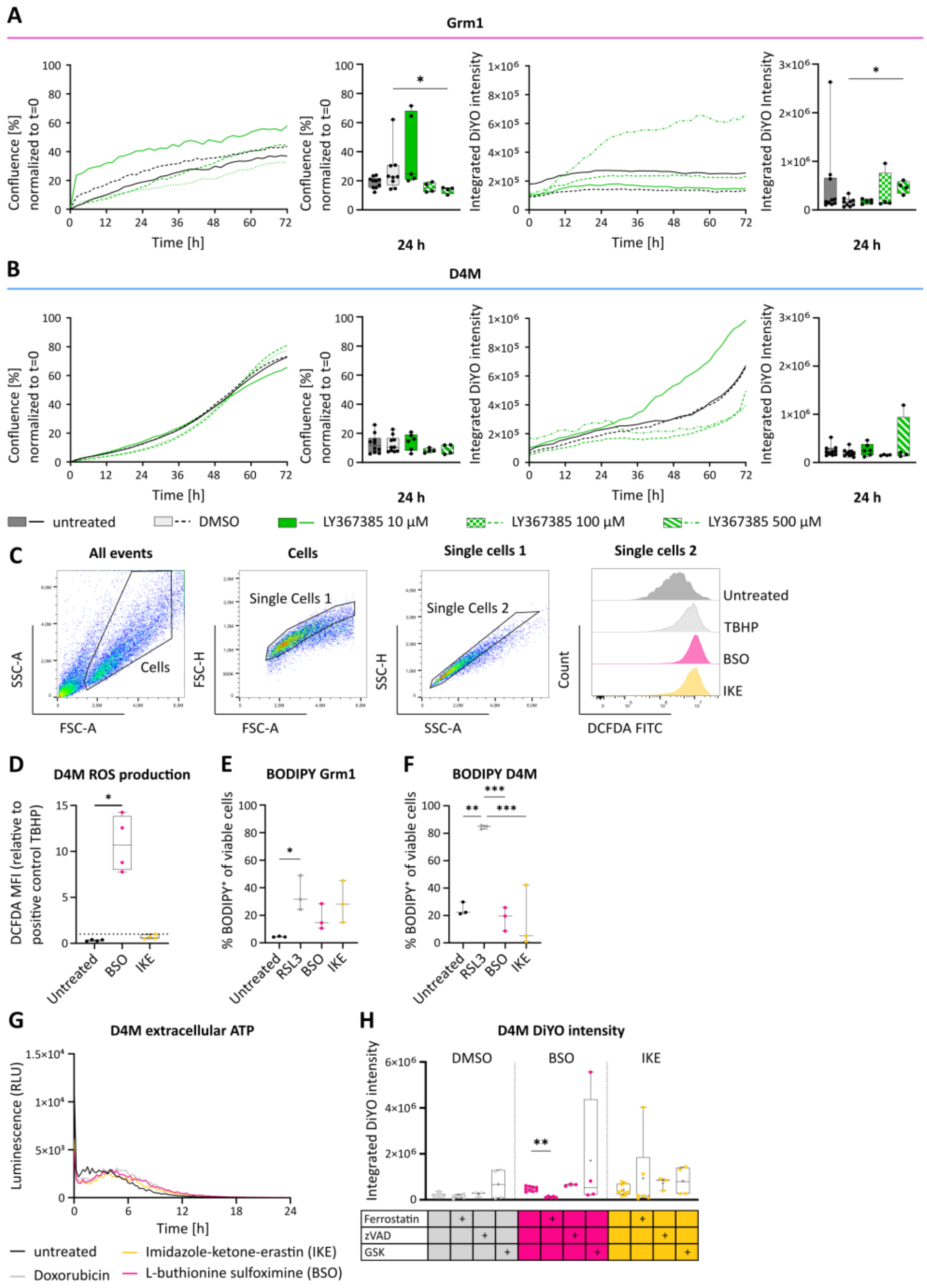


**Supplementary Figure S6. Grm1 cells react more sensitive to glutamate pathway and Grm1 inhibition than D4M cells. (A and B)** Cell confluence (normalized to the starting confluence at t = 0) and cell death (determined as DiYO intensity) of the cell lines **(A)** Grm1 and **(B)** D4M treated with the Grm1 inhibitor LY367385 (10, 100, and 500 µM), and controls (medium or 4% DMSO). The 24 h time point is displayed as whisker plots. Results from 5 independent experiments (n ≥ 4). **(C)** Gating strategy for flow cytometry analysis of Grm1 and D4M tumor cells. After excluding cellular debris, doublets, and dead cells, DCFDA+ cells were gated. Representative histogram for DCFDA expression of untreated, TBHP (250 µM), IKE (1 µM), and BSO (100 µM) treated Grm1 cells. **(D)** Intracellular reactive oxygen species (ROS) production in D4M cells after 24 h treatment with 100 µM BSO and 1 µM IKE (n ≥ 3). Lipid peroxidation of **(E)** Grm1 cells and **(F)** D4M cells after 1 h treatment with 100 µM BSO, 1 µM IKE, or 5 µM RSL3 were measured by BODIPY 581/591 C11 fluorescence (detected in FITC channel). Results from two independent experiments (n = 2). **(G)** Extracellular ATP release by D4M cells treated with 100 µM BSO, 1 µM IKE, or the positive control 5 µM doxorubicin was measured over 24 h using the RealTime-Glo™ Extracellular ATP Assay (n = 3). **(H)** D4M cells were treated with 1 µM IKE, 100 µM BSO, or 4% DMSO control together with 4 µM of the ferroptosis inhibitor Ferrostatin, 20 µM of the pyroptosis/apoptosis inhibitor zVAD-fmk, or 10 µM of the necroptosis inhibitor GSK'872. Results from three independent experiments (n ≥ 3). Statistical significance was determined using one-way analysis of variance, followed by Tukey's multiple comparison test, or Kruskal-Wallis test, followed by Dunn's multiple comparison test. Box and whisker plots showing all points, min to max. *p < 0.05; **p < 0.01; ***p < 0.001; ****p < 0.0001.

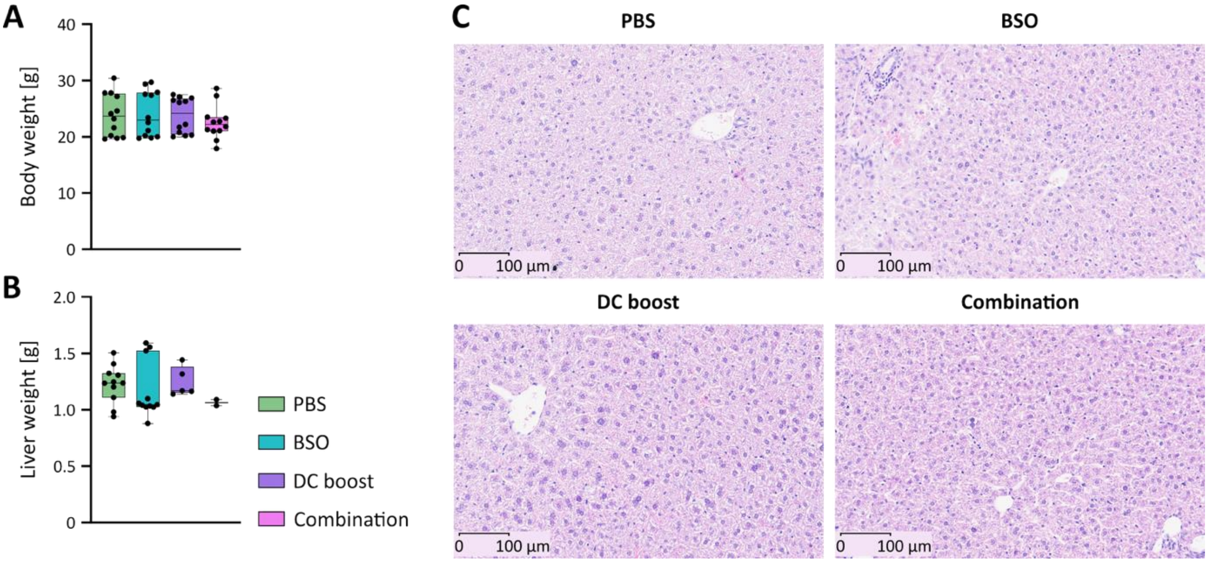


**Supplementary Figure S7. Mice treated with BSO show no liver toxicities after 3 weeks of treatment.** EPv mice at the transition from tumor-free (TF) to tumor-early (TE) stage were treated with glutamate-cysteine ligase (Gclc) inhibitor BSO and DC boost combination therapy over 3 weeks. **(A)** Body weight and **(B)** liver weights were documented at the end of treatment. **(C)** Representative images of liver H&E staining (bar = 100 µM). Results from three independent experiments are shown (n ≥ 2 mice/group). Statistical significance was determined using one-way analysis of variance followed by Tukey's multiple comparison test, or Kruskal-Wallis test followed by Dunn's multiple comparison test. Box and whisker plots showing all points, min to max. *p < 0.05; **p < 0.01; ***p < 0.001; ****p < 0.0001.

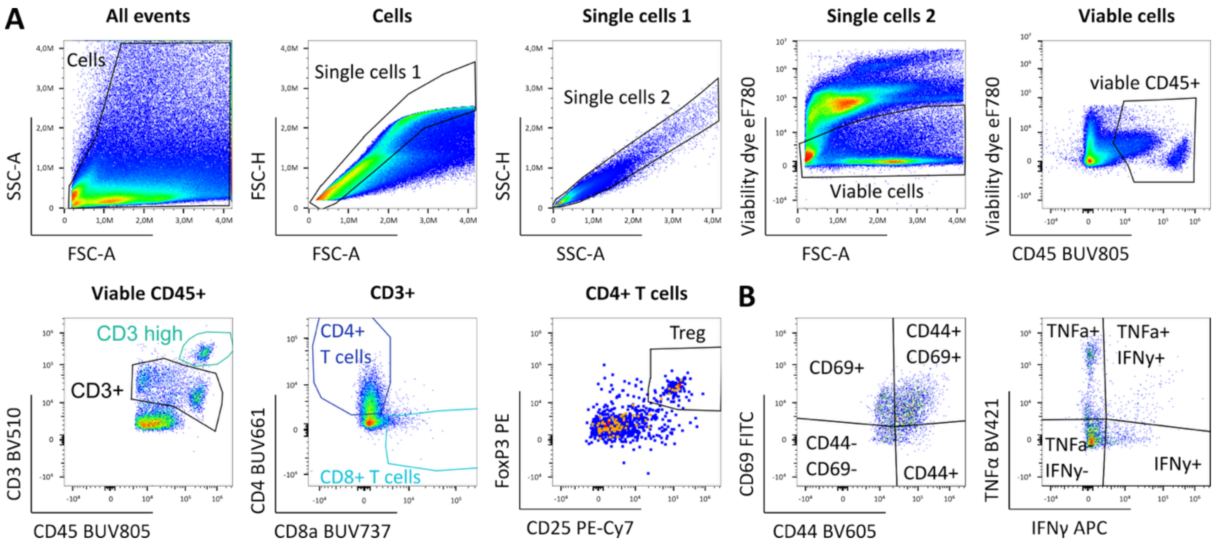


**Supplementary Figure S8. Gating strategy for T cell subsets in mouse and tumor ear skin.** EPv mice at the transition from tumor-free (TF) to tumor-early (TE) stage were treated with glutamate-cysteine ligase (Gclc) inhibitor BSO and DC boost combination therapy over 3 weeks. **(A)** Ear skin was enzymatically digested to obtain single cell suspensions. Gating strategy to identify T cell subpopulations in ear skin by flow cytometry. Cells are pre-gated on single, viable, CD45+cells, followed by $CD3^{+}$ and $CD3^{high}$ T cell gates. $CD3^{+}$ T cells were subdivided into $CD4^{+}$ and $CD8^{+}$ T cells, followed by gating for regulatory T cells ($CD25^{+}FoxP3^{+}$ Treg). **(B)** All T cell subsets were further analyzed for activation markers (CD69, CD44), and intracellular cytokine expression (TNFα, IFNγ).

**Supplementary Table T1:** Chemicals used for mass spectrometric analysis of carbon metabolites and organic acids.

| Reagent | Company / Source |
|---|---|
| Methanol (HPLC-grade) | Honeywell (Seelze, Germany) |
| Acetonitrile (HPLC-grade) | Honeywell (Seelze, Germany) |
| Water (HPLC-grade) | Honeywell (Seelze, Germany) |
| Triethylamine | Sigma-Aldrich (St. Louis, MO, USA) |
| Phenyl isothiocyanate | Sigma-Aldrich (St. Louis, MO, USA) |
| Acetic acid | Sigma-Aldrich (St. Louis, MO, USA) |
| Medronic acid | Sigma-Aldrich (St. Louis, MO, USA) |
| Ammonium acetate | Sigma-Aldrich (St. Louis, MO, USA) |
| EDTA | Sigma-Aldrich (St. Louis, MO, USA) |
| Reference standards of targeted intermediates of glycolysis and TCA cycle with their isotopically labeled analogs | Sigma-Aldrich (St. Louis, MO, USA) |
| Amino acid standards (A9906, asparagine, glutamine, kynurenine) | Sigma-Aldrich (St. Louis, MO, USA) |
| Stable isotope-labeled amino acid mix (96378) | Sigma-Aldrich (St. Louis, MO, USA) |
| Tryptophan-D5 | Provided by Toronto Research Chemicals (Toronto, Ontario, Canada) |

**Supplementary Table T2:** Compound specific ion transitions and corresponding experimental parameters for mass spectrometric analysis of carbon metabolites and organic acids.

| Compound | Q1 *m/z* | Q3 *m/z* | Internal Standard |
|---|---|---|---|
| 2.3-Bisphosphoglycerate | 265.1 | 167 | Phosphoenolpyruvic acid-$^{13}C_3$ |
| ATP | 506 | 159 | Phosphoenolpyruvic acid-$^{13}C_3$ |
| 2-Ketoglutaric acid | 145 | 101.1 | Fumaric acid-$^{13}C_4$ |
| 2-Phosphoglycerate | 185 | 79 | Phosphoenolpyruvic acid-$^{13}C_3$ |
| 3-Phosphoglycerate | 185 | 97 | Phosphoenolpyruvic acid-$^{13}C_3$ |
| Citric acid | 191.1 | 111.1 | Phosphoenolpyruvic acid-$^{13}C_3$ |
| Dihydroxyacetone phosphate | 169 | 97 | Phosphoenolpyruvic acid-$^{13}C_3$ |
| Fructose-1.6-bisphosphate | 339 | 97 | Phosphoenolpyruvic acid-$^{13}C_3$ |
| Fructose-6-phosphate/ glucose-6-phosphate | 259 | 97 | Phosphoenolpyruvic acid-$^{13}C_3$ |
| Fumaric acid | 115 | 71 | Fumaric acid-$^{13}C_4$ |
| Glucose | 179 | 59 | Lactic acid-$^{13}C_3$ |
| Lactic acid | 89.1 | 43.1 | Lactic acid-$^{13}C_3$ |
| Malic acid | 133 | 115 | Fumaric acid-$^{13}C_4$ |
| Oxaloacetic acid | 131.1 | 87.1 | Fumaric acid-$^{13}C_4$ |
| Phosphoenolpyruvic acid | 166.9 | 79 | Phosphoenolpyruvic acid-$^{13}C_3$ |
| Pyruvic acid | 87.1 | 43.3 | Pyruvic acid-$^{13}C_3$ |
| Succinic acid | 117 | 73 | Fumaric acid-$^{13}C_4$ |
| Lactic acid-$^{13}C_3$ | 92 | 45.2 | |
| Fumaric acid-$^{13}C_4$ | 119 | 74 | |
| Pyruvic acid-$^{13}C_3$ | 90 | 45.2 | |
| Phosphoenolpyruvic acid-$^{13}C_3$ | 170 | 79 | |

**Supplementary Table T3:** Precursor-to-product ion transitions for mass spectrometric analysis of amino acids and biogenic amines.

| Target | Q1 (*m/z*) | Q3 (*m/z*) | Internal Standard |
|---|---|---|---|
| Alanine | 225 | 44 | Alanine-$^{13}C_3{}^{15}N$ |
| Arginine | 310 | 217 | Arginine-$^{13}C_6$ |
| Aspartic acid | 269 | 116 | Aspartic acid-$^{13}C_4$ |
| Glutamic acid | 283 | 130 | Glutamic acid-$^{13}C_5$ |
| Glycine | 211 | 76 | Glycine-$^{13}C_2{}^{15}N$ |
| Histidine | 291 | 110 | Histidine-$^{13}C_6$ |
| Leucine | 267 | 43 | Leucine-$^{13}C_6{}^{15}N$ |
| Isoleucine | 267 | 69 | Isoleucine-$^{13}C_6{}^{15}N$ |
| Lysine | 417 | 324 | Lysine-$^{13}C_6$ |
| Methionine | 285 | 104 | Methionine-$^{13}C_5{}^{15}N$ |
| Phenylalanine | 301 | 120 | Phenylalanine-$^{13}C_6$ |
| Proline | 251 | 70 | Proline-$^{13}C_5$ |
| Serine | 241 | 60 | Serine-$^{13}C_3{}^{15}N$ |
| Threonine | 255 | 74 | Threonine-$^{13}C_4$ |
| Tryptophan | 340 | 188 | Tryptophan-$D_5$ |
| Tyrosine | 317 | 136 | Tyrosine-$^{13}C_6$ |
| Valine | 253 | 72 | Valine-$^{13}C_5$ |
| Asparagine | 268 | 87 | Serine-$^{13}C_3{}^{15}N$ |
| Carnosine | 362 | 110 | Arginine-$^{13}C_6$ |
| Citrulline | 311 | 113 | Serine-$^{13}C_3{}^{15}N$ |
| Glutamine | 282 | 130 | Serine-$^{13}C_3{}^{15}N$ |
| Kynurenine | 344 | 146 | Tryptophan-$D_5$ |
| Ornithine | 403 | 310 | Methionine-$^{13}C_5{}^{15}N$ |
| c4-OH-Pro | 267 | 68 | Arginine-$^{13}C_6$ |
| Alanine-$^{13}C_3{}^{15}N$ | 229 | 47 | |
| Arginine-$^{13}C_6$ | 316 | 223 | |
| Aspartic acid-$^{13}C_4$ | 273 | 120 | |
| Glycine-$^{13}C_2{}^{15}N$ | 214 | 79 | |
| Histidine-$^{13}C_6$ | 297 | 115 | |
| Leucine-$^{13}C_6{}^{15}N$ | 274 | 92 | |
| Isoleucine-$^{13}C_6{}^{15}N$ | 423 | 330 | |
| Lysine-$^{13}C_6$ | 307 | 126 | |
| Phenylalanine-$^{13}C_6$ | 256 | 74 | |
| Proline-$^{13}C_5$ | 245 | 63 | |
| Serine-$^{13}C_3{}^{15}N$ | 259 | 63 | |
| Threonine-$^{13}C_4$ | 259 | 77 | |
| Tyrosine-$^{13}C_6$ | 323 | 142 | |
| Valine-$^{13}C_5$ | 258 | 76 | |
| Glutamic acid-$^{13}C_5$ | 288 | 135 | |
| Methionine-$^{13}C_5{}^{15}N$ | 291 | 109 | |
| Tryptophan-$D_5$ | 345 | 193 | |

**Supplementary Table T4:** Antibodies used for flow cytometry.

| Antibody (Fluorochrome) | Clone | Manufacturer | Identifier |
|---|---|---|---|
| CCR2 BV711 | 475301 | 747964 | 747964 |
| CCR7 APC | 4B12 | Biolegend | 120108 |
| CD103 BB790 | M290 | BD | Custom-made |
| CD11b BUV496 | M1/70 | BD | 749864 |
| CD11c BUV395 | N418 | BD | 744180 |
| CD172a FITC | P84 | Biolegend | 144006 |
| CD19 BB660 | 1D3 | BD | Custom-made |
| CD207 A546 | 929F3.01 | Dendritics | DDX0362A546-100 |
| CD207 PE | 929F3.01 | self-conjugated | |
| CD24 BV786 | M1/69 | 744470 | 744470 |
| CD25 PE-Cy7 | PC61 | BioLedgend | 102015 |
| CD3 BV510 | 17A2 | BioLedgend | 100234 |
| CD3e PE-Cy5 | 145-2C11 | BD | 553065 |
| CD4 BUV661 | RM4-5 | BD Bioscience | 741461 |
| CD40 BB700 | 3/23 | BD | 742136 |
| CD44 BV605 | IM7 | BioLedgend | 103047 |
| CD45 BUV805 | 30-F11 | BD Bioscience | 748370 |
| CD64 PE-Cy7 | X54 | Biolegend | 139314 |
| CD69 FITC | H1.2F3 | BioLedgend | 104506 |
| CD8a BUV737 | 53-6.7 | BD Bioscience | 612759 |
| CTLA-4 (CD152) APC-R700 | UC10-4F10-11 | BD Bioscience | 565778 |
| F4/80 PE-Dazzle594 | BM8 | Biolegend | 123146 |
| FoxP3 PE | FJK-16s | invitrogen | 12-5773-82 |
| IFNy APC | XMG1.2 | BioLedgend | 505810 |
| Ly-6C BV421 | HK1.4 | Biolegend | 128031 |
| Ly-6G BV570 | 1A8 | Biolegend | Custom-made |
| MHC class II BV480 | M5/114 | BD | 566086 |
| NK1.1 BB630 | PK136 | BD | Custom-made |
| PD-1 (CD279) PE-Dazzle | RMP1-30 | BioLedgend | 109116 |
| PD-L1 BUV615 | MIH5 | BD | 752339 |
| PD-L2 R718 | TY25 | 752227 | 752227 |
| pDCA-1 BV750 | 927 | 747608 | 747608 |
| TNFa BV421 | MP6-XT22 | BioLedgend | 506327 |
| XCR1 BV650 | ZET | 148220 | 148220 |

**Supplementary Table T5:** Cell lines.

| Cell line | Genotype | Culture medium | Company / Source |
|---|---|---|---|
| D4M.3A | $BRAF^{V600E}$ mutant cell line from Tyr::CreER,BRAFCA, Pten $^{lox/lox}$ mice | High Glucose DMEM (Sigma-Aldrich) +5 % FCS +1 % P/S | Kindly provided by Constance Brinckerhoff, New Hampshire |
| Grm1 | Non-pigmented melanoma cells expressing Grm1 derived from tumors of the tg(Grm1)EPv mice | High Glucose DMEM (Sigma-Aldrich) +10 % FCS +2 % P/S | Kindly provided by Anja Bosserhoff, Erlangen |

**Supplementary Table T6:** Primers for gene expression analysis.

| Gene id/Protein | Code or Sequence | Manufacturer |
|---|---|---|
| Mouse Grm1 | Mm00810231_s1 | Thermo Fisher |
| TATA-binding protein (tbp) | fw: CTTCGTGCAAGAAATGCTGAA<br>(10 µM)<br>rv: TGTCCGTGGCTCTCTTATTCTCA<br>(10 µM)<br>Probe (5'FAM p3'TAMRA):<br>TCCCAAGCGATTTGCTGCAGTCATC<br>(5 µM) | Microsynth |

**Supplementary Table T7:** Reagents/antibodies for in vivo treatments.

| Reagent/Antibody | Clone | Route of Administration / Amount per Mouse | Company / Source |
|---|---|---|---|
| Recombinant human Flt3 ligand | N/A | i.t. / 10 µg | Kindly provided by Tibor Keler, Celldex Therapeutics, Hampton, NJ, USA |
| Anti-mouse CD40 agonistic mAb | FGK45 | i.d. / 25 µg | Produced in-house (kindly provided by Louis Boon (JJPBio)) |
| Poly I:C | N/A | i.t. / 25 µg | Sigma-Aldrich |
| L-Buthionine-(S,R)-sulfoximine (L-BSO) | N/A | i.p. / 1,11 mg | MedChemExpress (MCE) |
